# Genome-wide mapping of transcriptional enhancer candidates using DNA and chromatin features in maize

**DOI:** 10.1186/s13059-017-1273-4

**Published:** 2017-07-21

**Authors:** Rurika Oka, Johan Zicola, Blaise Weber, Sarah N. Anderson, Charlie Hodgman, Jonathan I. Gent, Jan-Jaap Wesselink, Nathan M. Springer, Huub C. J. Hoefsloot, Franziska Turck, Maike Stam

**Affiliations:** 1 Swammerdam Institute for Life Sciences, Universiteit van Amsterdam, Science Park 904, 1098 XH Amsterdam, The Netherlands; 2 Department Plant Developmental Biology, Max Planck Institute for Plant Breeding Research, Carl-von-Linné-Weg 10, 50829 Köln, Germany; 3 Department of Plant Biology, University of Minnesota, 40 Gortner Laboratory, 1479 Gortner Avenue, St. Paul, MN 55108 USA; 4 Centre for Plant Integrative Biology, School of Biosciences, University of Nottingham, Sutton Bonington, LE12 5RD UK; 5 Department of Plant Biology, University of Georgia, Athens, GA 30602 USA; 6 Diagenode, S.A, Rue du Bois Saint-Jean 3, 4102 Liège, Belgium

**Keywords:** Transcriptional enhancer, Gene regulation, Chromatin accessibility, Histone acetylation, DNA methylation, *Zea mays*

## Abstract

**Background:**

While most cells in multicellular organisms carry the same genetic information, in each cell type only a subset of genes is being transcribed. Such differentiation in gene expression depends, for a large part, on the activation and repression of regulatory sequences, including transcriptional enhancers. Transcriptional enhancers can be located tens of kilobases from their target genes, but display characteristic chromatin and DNA features, allowing their identification by genome-wide profiling. Here we show that integration of chromatin characteristics can be applied to predict distal enhancer candidates in *Zea mays*, thereby providing a basis for a better understanding of gene regulation in this important crop plant.

**Result:**

To predict transcriptional enhancers in the crop plant maize (*Zea mays* L. ssp. mays), we integrated available genome-wide DNA methylation data with newly generated maps for chromatin accessibility and histone 3 lysine 9 acetylation (H3K9ac) enrichment in young seedling and husk tissue. Approximately 1500 intergenic regions, displaying low DNA methylation, high chromatin accessibility and H3K9ac enrichment, were classified as enhancer candidates. Based on their chromatin profiles, candidate sequences can be classified into four subcategories. Tissue-specificity of enhancer candidates is defined based on the tissues in which they are identified and putative target genes are assigned based on tissue-specific expression patterns of flanking genes.

**Conclusions:**

Our method identifies three previously identified distal enhancers in maize, validating the new set of enhancer candidates and enlarging the toolbox for the functional characterization of gene regulation in the highly repetitive maize genome.

**Electronic supplementary material:**

The online version of this article (doi:10.1186/s13059-017-1273-4) contains supplementary material, which is available to authorized users.

## Background

Successful differentiation of zygotes into different cell types that make up a complex multicellular organism requires flexibility to respond to environmental cues, but also a tight control of gene expression during developmental processes. Regulation of gene expression, among others, depends on a complex network of sequence-specific transcription factors (TFs) as well as protein factors that can read or write chromatin modifications [1, 2]. In addition, gene expression regulation depends on genetic information encoded within *cis*-regulatory regions such as transcriptional promoters and enhancers, which contain multiple TF binding sites and display particular DNA and chromatin features [3]. In the last decade, genome-wide approaches in animals have identified thousands of enhancers (see e.g. [4]). Mutations in enhancers are known to cause developmental defects, cancer or other diseases [5–8], emphasising the crucial role of enhancers in gene expression regulation. High-throughput genome-wide enhancer identification in plant species only started recently and only a small number of enhancers were thoroughly studied in plant species [9, 10], including enhancers for *booster1* (*b1*) [11, 12], *teosinte branched1* (*tb1*) [13, 14], *pericarp color1* (*p1*) [15] in maize, *Block C* for *FLOWERING LOCUS T* in *Arabidopsis thaliana* (Arabidopsis) [16, 17] and the enhancers for the chlorophyll *a/b*-binding protein gene *AB80* and *pea plastocyanin* gene in *Pisum sativum* [18, 19]. So far, few genome-wide approaches to identify *cis-*regulatory sequences in plants have been reported, i.e. in Arabidopsis, *Oryza sativa* (rice) and maize [20–22]. Although multiple studies in plants reported genome-wide profiles for different chromatin features, only one, in Arabidopsis, aimed at discovering enhancers [20].

Enhancers can be located upstream or downstream of their target genes and physically interact with their target genes to regulate gene expression [23, 24]. They are typically short DNA sequences of 50–1000 bps that are bound by TFs and characterised by an accessible chromatin structure, especially when they are actively involved in regulating gene expression [25, 26]. Based on extensive studies in animals, active enhancers are associated with low DNA methylation and histone modifications such as acetylation of lysines 9, 14 and 27 of histone H3 (H3K9ac, H3K14ac and H3K27ac) [27–30]. Mono-methylation of lysine 4 of histone H3 (H3K4me1) is enriched at enhancers regardless of their activity [27, 28]. Low DNA methylation has been reported to positively correlate with enhancer activity and also used to predict enhancers [29, 31]. Although limited data are currently available, similar DNA and chromatin features were observed at known plant enhancers, indicating that these marks may, at least partially, be conserved between animals and plants [9].

Another feature reported for animal enhancers is bi-directional transcription, producing so-called enhancer RNA (eRNA). eRNA expression levels positively correlate with enhancer target gene expression levels [4, 32], which can help to link enhancers to their target genes. The function of eRNAs is not yet clear, but some of them have been reported to play a role in the recruitment of TFs to enhancers or in the formation of enhancer–promoter interactions [33, 34].

The purpose of this study was a genome-wide identification of active intergenic enhancers in maize and to find their most likely target genes by integrating tissue-specific chromatin features and differential gene expression levels. To do so, we identified regions with low DNA methylation levels using published bisulphite-sequencing (BS-seq) data [35] and measured chromatin accessibility using DNase-seq, H3K9 acetylation using chromatin immunoprecipitation sequencing (ChIP-seq) and differential expression using RNA sequencing (RNA-seq) in V2 stage inner stem tissue (V2-IST) and husk tissue. We identified approximately 1500 intergenic enhancer candidates and defined their tissue-specificity based on the presence or absence of DNase I hypersensitivity and H3K9ac enrichment signals. Our pipeline was validated by the detection of three previously identified (putative) enhancers, regulating the expression of *b1*, *bx1* and *tb1*.

## Results

### Selection of H3K9ac as best suited histone modification to identify active enhancers in maize

**Fig. 1:**
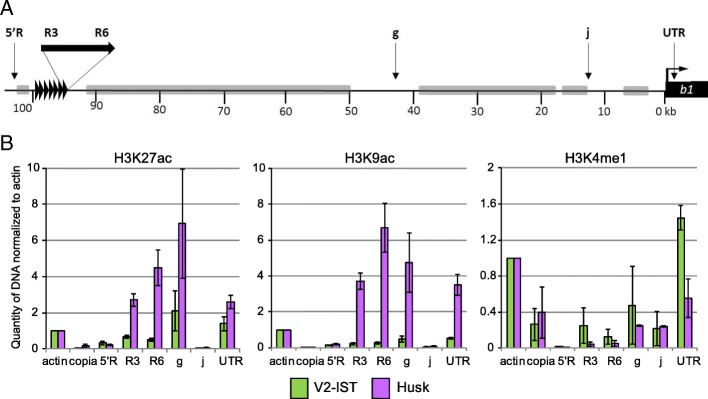
ChIP-quantitative polymerase chain reaction (qPCR) analysis at *b1* for H3K27ac, H3K9ac and H3K4me1. **a** *Schematic representation* of the *b1* locus. *Vertical arrows* with *letters* indicate the regions examined by ChIP-qPCR. The *b1* hepta-repeat enhancer is indicated with *seven black triangles*, the *b1* coding region by a *black box* and the TSS by a bent *arrow. Grey bars* represent TEs and other repetitive sequences. **b** Enrichment of H3K27ac, H3K9ac and H3K4me1 at the *b1* locus relative to the enrichment at the maize *actin 1* locus (*actin*). *Error bars* represent the standard error of the mean for two (H3K9ac, H3K4me1) or three (H3K27ac) biological replicates

In mammals, several histone modifications such as H3K27ac, H3K9ac and H3K4me1 were shown to mark active enhancers [27, 28, 30]. To define which of these histone modifications indicate best active enhancers in maize, we examined the enrichment of H3K27ac, H3K9ac and H3K4me1 at the hepta-repeat enhancer and other *cis-*regulatory sequences present at the *B-I* allele of the *b1* gene. ChIP was performed on inner stem tissue from V5 maize seedlings (V5-IST) and husk tissue. The hepta-repeat enhancer of *B-I*, located 100 kb upstream of the *b1* transcription start site (TSS), is inactive in V5-IST and active in husk leaves [36]. Previously, the hepta-repeat enhancer and regulatory sequences ~45 kb upstream of *b1* were shown to be enriched with H3K9K14ac when active [36]. The results presented here (Fig. 1) indicated that the enrichment in both H3K9ac and H3K27ac was significantly higher in husk compared to V5-IST at the hepta-repeat enhancer (R3 and R6), ~45 kb upstream regulatory sequences (g) and the untranslated 5’ region of *b1* (UTR). Based on these results, both H3K9ac and H3K27ac appeared to mark active enhancers. In contrast, H3K4me1 enrichment levels were relatively low throughout the intergenic *b1* region in both V5-IST and husk tissues. In addition, at the coding region, H3K4me1 enrichment levels were higher in low *b1* expressing V5-IST than in high expressing husk tissue. Therefore, in contrast to animal systems [27, 37], H3K4me1 is probably not suited to identify enhancers in maize. Since the enrichment at the enhancer region in husk relative to V5-IST tissue was highest for H3K9ac, we chose this histone modification to identify active enhancers genome-wide.

### An integrated pipeline to identify tissue-specific enhancers in maize

DNase-seq, H3K9ac ChIP-seq and RNA-seq experiments were carried out in two tissues, V2-IST and husk, isolated from the reference inbred line B73 (Additional file 1: Figure S1). These tissues were selected to identify tissue-specific as well as developmental stage-specific enhancers. Our study included material grown at two different locations (DNase-seq and H3K9ac ChIP-seq were performed at the Max Planck Institute for Plant Breeding Research and the University of Amsterdam, respectively); therefore, we performed RNA-seq experiments for each tissue in six biological replicates, three per location. Comparison of gene expression levels between replicates in reads per kilobase of transcript per million mapped reads (RPKM) revealed high correlations among replicates between the two locations (Additional file 1: Figure S2). This high correlation between replicates and locations indicated the data were comparable and implied that the chromatin states of the plants from both locations were similar. Gene expression levels and significant differential expression levels were calculated, taking the variability among six replicates into account. The genes determined as significantly differentially expressed thus showed statistically significant differences in their expression levels at both locations.

**Fig. 2:**
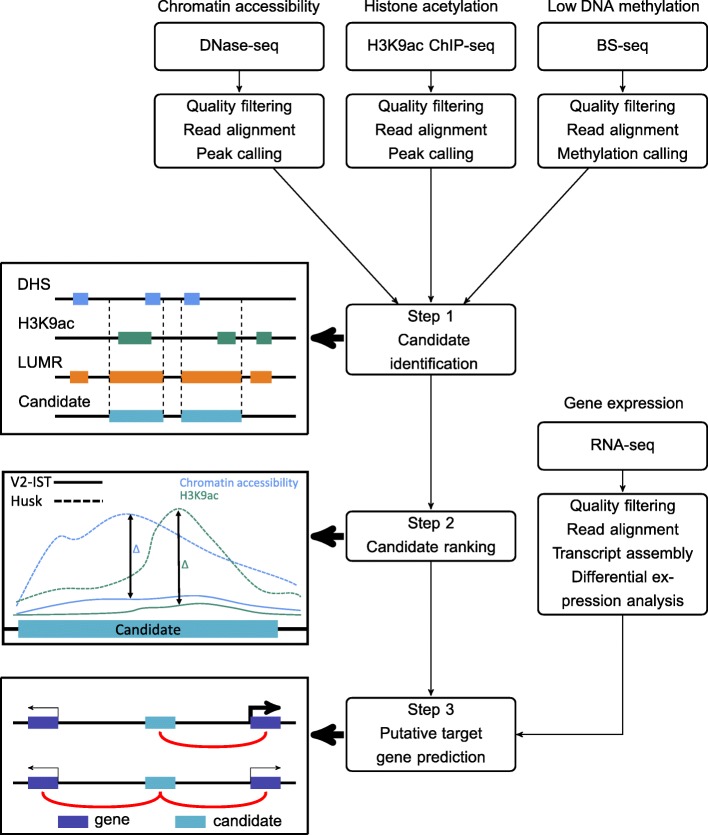
Overall workflow of this study. First, chromatin accessibility data from DNase-seq, H3K9ac enrichment data from ChIP-seq and DNA methylation data from BS-seq were analysed individually. Second, the data on accessible regions, H3K9ac-enriched regions and low DNA methylated regions were integrated to predict enhancers. Third, the enhancer candidates were ranked based on signal intensity differences of the chromatin accessibility and H3K9ac enrichment data between V2-IST and husk tissue. Finally, enhancer candidates were linked to their putative target genes based on their tissue specificity and on the differential expression of flanking genes determined by RNA-seq data. For shared candidates, adjacent genes being expressed in both tissues were associated

After pre-processing of the data, our enhancer prediction pipeline consisted of three steps of data integration (Fig. 2). First, enriched chromatin or DNA features were identified for three genome-wide datasets. In addition to calling DNase-seq and H3K9ac ChIP-seq peaks from our own datasets, we identified low and unmethylated DNA regions (LUMRs) by re-analysing published BS-seq data [35]. By taking an overlap between the three datasets, regions displaying all three features were selected as enhancer candidate regions. We focused on intergenic enhancer candidates, excluding promoter regions, as chromatin profiles of enhancers located in proximity of and within coding regions are more likely to overlap with chromatin profiles of genic regions, making it difficult to disentangle the underlying regulatory regions. Enhancer candidates predicted in only one tissue were defined as tissue-specific candidates. Transposable elements (TEs) were included in our analysis as some of them had been shown or suggested to act as enhancers in maize and other organisms [13, 38]. The second step involved determining the degree of tissue-specificity of the candidates identified in both tissues by ranking the candidates based on signal intensity differences between the two tissues. This was done for both chromatin accessibility and H3K9ac enrichment, followed by summing the ranks and re-ranking. The last step assigned target genes to enhancer candidates, assuming that enhancers most likely regulate genes located directly upstream or downstream and that gene expression and active chromatin marks at enhancers are positively correlated.

### Distribution of chromatin features in the uniquely mappable part of the maize genome

**Fig. 3:**
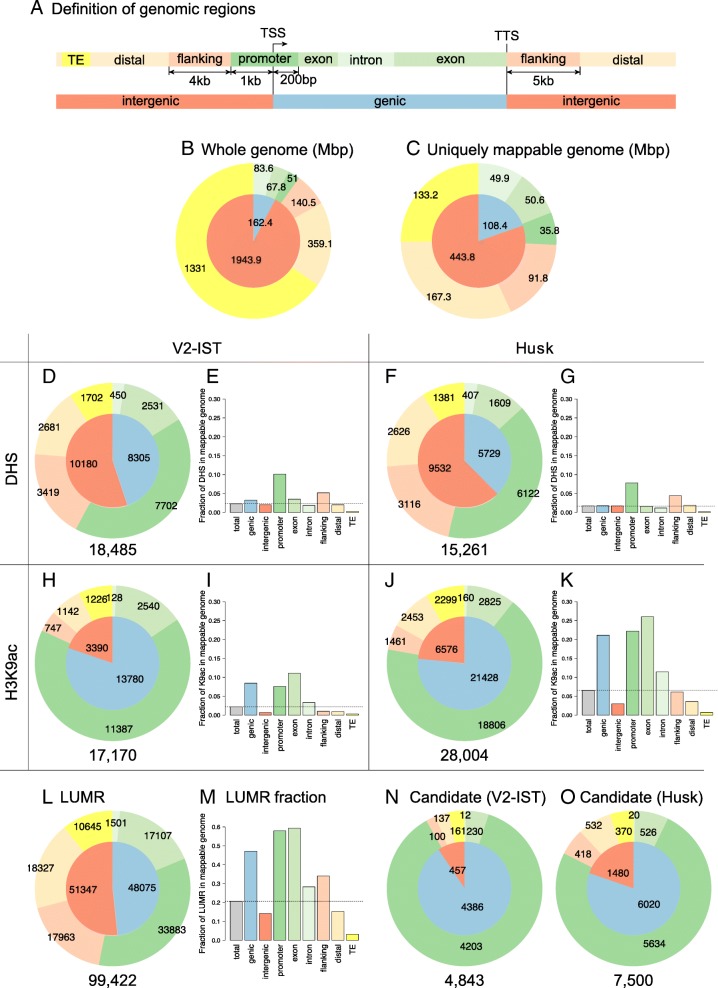
Genomic composition and distribution of features. **a** Definition of genomic regions. Promoters are defined from 1 kb upstream to 200 bp downstream from the TSSs, flanking regions are 4 kb upstream from the promoters and 5 kb downstream from the TTSs. *TE* transposable elements, *distal* intergenic regions that are more than 5 kb away from genic regions and are not TEs. **b** Composition of the entire maize genome according to AGPv4 and (**c**) the uniquely mappable genome. Distribution of (**d**, **f**) DHSs, (**h**, **j**) H3K9ac, (**l**) LUMRs and (**n**, **o**) enhancer candidates over the different genomic regions, and (**e**, **g**, **i**, **k**, **m**) the fractions (Mbp/Mbp, from 0 to 1, *y-axes*) the different features (*x-axes*) occupy at the various genomic regions in the uniquely mappable genome. The *grey bars* indicate the fraction of overall occupancy in the uniquely mappable genome.

To identify chromatin accessibility, H3K9ac enrichment, and low DNA methylation within the genome, we partitioned the genic and intergenic regions of the genome in six sub-categories: promoters; exons; introns; flanking and distal intergenic regions; and TEs (Fig. 3a). Gene annotations were taken from the maize B73 annotation version 4 (AGPv4 assembly [39]) from Ensembl Plants [40]. Only intergenic TEs were considered in our study; TEs present in introns were counted as ‘introns’. Promoter regions were defined as 1 kb upstream to 200 bp downstream from the TSS, therefore including the first nucleosome downstream of the TSS. The composition of the B73 maize genome was quantified by counting the numbers of mega bases in each genomic region (Fig. 3b). Since 85% of the maize genome is highly repetitive [41], an important fraction of the next-generation sequencing reads could not be mapped uniquely (Additional file 1: Table S1), which prevented enhancer identification in repetitive genomic regions. We determined the uniquely mappable parts of the genome by performing an all-against-all alignment for theoretical 93 bp single-end reads, allowing a maximum of two mismatches using the Uniqueome pipeline [42], which estimates the fraction of uniquely mapped reads for each nucleotide (Fig. 3c). In the uniquely mappable genome, the proportion of TEs was reduced to approximately one-quarter of the assembled genome.

### 9212 intergenic DHSs are potential *cis*-regulatory elements

DNase I hypersensitive sites (DHSs) are genomic regions that are more sensitive to DNase I endonuclease activity compared with flanking regions due to a lower nucleosome density [43]. The mapping of DHSs by DNase-seq is a powerful approach to identify *cis-*regulatory regions, including enhancers, and has been used in many organisms including plants [20, 25, 44–46]. DNase-seq experiments were performed in two biological replicates for both V2-IST and husk tissue (Additional file 1: Table S1). To take the intrinsic digestion bias of DNase I into account, we also included a control sample generated by digesting B73 genomic DNA (gDNA) with DNase I. After mapping the reads obtained from each library, DHSs were identified for each library using MACS2 peak calling [47].

Data reproducibility between biological replicates was examined by counting the number of overlapping DHSs identified for all the possible combinations of replicates (Additional file 1: Table S2). This comparison showed that 54–92% of DHSs overlapped by at least 1 bp between replicates. The overlap between the two V2-IST replicates was the lowest (54% of the 35,906 V2-IST_2 peaks were overlapping with the 21,309 V2-IST_1 peaks) as 1.5 times more peaks were identified in the V2-IST_2 sample. The overlap between peaks identified in V2-IST and in husk samples appeared quite large (e.g. 80% of the peaks identified in V2-IST_1 were also observed in Husk_1), indicating that most DHSs are not tissue-specific. To select for high confidence DHSs in both V2-IST and husk tissue, only DHSs overlapping by at least 70% of their lengths between replicates were kept for further analysis. For signal intensity analysis, the reads in all biological replicates were pooled per tissue to estimate the overall coverage of the reads.

**Fig. 4:**
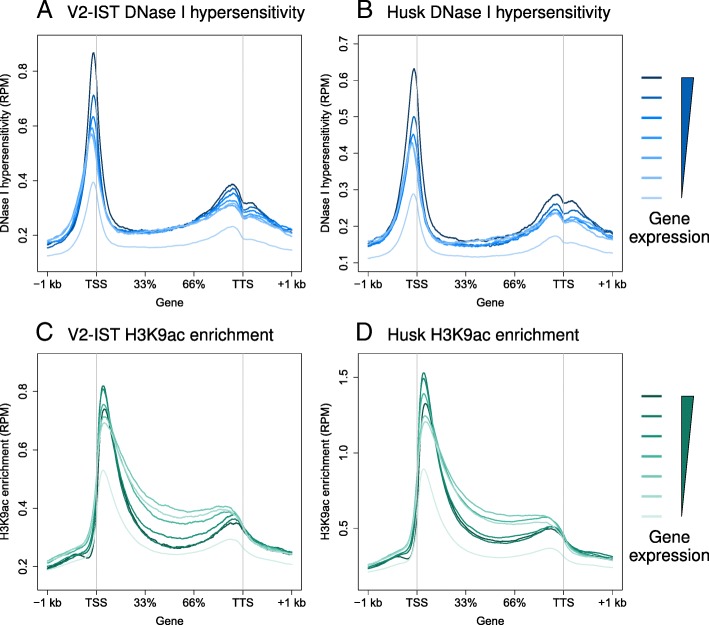
Average DNase I hypersensitivity and H3K9ac enrichment at genic regions. Average signal (in RPM) for DNase I hypersensitivity in (**a**) V2-IST and (**b**) husk, and for H3K9ac enrichment in (**c**) V2-IST and (**d**) husk at genes and their 1-kb flanking regions. Genes were binned based on their expression levels, from no expression (*light colour*) to high expression (*dark colour*): the lowest expression level bin contains all genes with an expression lower than 1 RPKM. The thresholds (in RPKM) are at 1.94, 4.17, 8.58, 16.64 and 36.28 for V2-IST and 1.88, 4.00, 8.34, 15.83 and 32.99 for husk tissue

We correlated DNase I hypersensitivity and gene expression levels in gene bodies and their immediate 1 kb flanking regions for additional validation of the dataset. For each tissue, genes were binned according to their gene expression levels and the average DNase I hypersensitivity, measured in number of read counts per million mapped reads (RPM), was calculated for each bin using bwtools [48] (Fig. 4a and b). A positive correlation between expression levels and DNase-seq coverage over genic regions was observed, especially directly upstream of the TSSs and transcription termination sites (TTSs). Chromatin at gene bodies was rather inaccessible among the gradient of gene expression. Presence of DHSs at TSSs and a positive correlation with expression levels observed in our dataset confirm previous observations in both animals and plants [21, 26, 49–51].

The number of DHSs per genomic region was counted to examine their fraction per genomic region (Fig. 3d, f). When comparing the distributions of DHSs to a randomised distribution within the mappable genome (Additional file 1: Figure S3A and B), we observed a clear over-representation of DHSs at promoters (*p* value < 0.001; permutation test). Still, 43% of DHSs, in total 9212 out of 21,445, were in intergenic regions excluding promoters (Fig. 3d, f): 7802 in V2-IST, 7123 in husk and 5130 shared between both tissues (Table 1A). In addition, the fraction of the genome scored as DHS (in Mbp) was calculated for each genomic category. In total, DHSs occupied about 2% of the mappable genome in both tissues (Fig. 3e, g). DHSs occupied 10% and 8% of the total mappable promoter regions in V2-IST and husk, respectively.

**Table 1:** Intergenic regions of interest

Tissue	All	Overlapping with LUMRs	Overlapping with H3K9ac
(A) Number of intergenic DHSs			
V2-IST	7802	7653 (98.1%)	344 (4.4%)
Husk	7123	6997 (97.4%)	1505 (21.1%)
Common	5130	5013 (97.7%)	202 (3.9%)
Total	9212	9057 (98.3%)	2030 (22.0%)
(B) Number of intergenic H3K9ac enriched regions peaks			
Tissue	All	Overlapping with LUMRs	Overlapping with DHSs
V2-IST	3115	3093 (99.3%)	323 (10.4%)
Husk	6213	6170 (99.3%)	998 (16.1%)
Common	2668	2652 (99.4%)	184 (6.9%)
Total	6511	6466 (99.3%)	1454 (22.3%)

(A) DHSs and (B) H3K9ac-enriched regions and number (and percentage in parentheses) of these regions overlapping with other features (LUMRs, H3K9ac or DHSs) in each tissue (V2-IST and Husk), common to both tissues (Common) and the sum of the tissues (Total)

### ChIP-seq identifies 6511 intergenic H3K9ac-enriched regions

ChIP-seq H3K9ac data were obtained from two and three biological replicates for V2-IST and husk tissue, respectively. The reads were aligned to the AGPv4 B73 reference genome and H3K9ac-enriched regions were identified, taking the input sample into account, by peak calling for each replicate using MACS2 [47].

To examine the reproducibility between replicates, overlapping H3K9ac-enriched regions were counted for all replicate combinations, showing 62–96% overlap within a tissue (Additional file 1: Table S3). As for the DNase-seq data, H3K9ac-enriched regions with an overlap in length of at least 70% between all replicates were kept for further analysis and reads in replicates were pooled for coverage calculation in each tissue. We correlated gene expression levels with H3K9ac enrichment levels across gene bodies and their 1-kb flanking regions (Fig. 4c, d) and observed a peak of H3K9ac enrichment immediately after the TSS and increased levels across the gene bodies compared to gene flanking regions. At the TSS peak region, gene expression and H3K9ac levels showed a parabolic correlation, showing saturation for higher bins and signal reduction for the highest one. In gene bodies, H3K9ac was lower for the three highest bins than for the three following bins. Previous studies in yeast and maize have reported a genome-wide loss of nucleosomes at highly expressed genes [26, 52]. Reduced nucleosome levels could explain the reduction in H3K9ac observed at highly expressed maize genes. Correlations between enrichment levels of H3K9ac 3’ of the TSS and gene expression levels have been previously reported [30, 53, 54]. Our data suggest that H3K9ac enrichment levels reached saturation for genes with high expression levels.

To estimate the number of potential intergenic enhancer candidates from the H3K9ac data sets, the genomic distribution of H3K9ac-enriched regions was examined by counting the numbers of H3K9ac-enriched regions in the different types of genomic regions (Fig. 3a, h, j). As seen for DHSs, a clear over-representation of H3K9ac-enriched regions at promoters was observed when compared with a randomised distribution (*p* value < 0.001; permutation test, Additional file 1: Figure S3C and D). In both tissues, nearly 70% of all H3K9ac-enriched regions located at promoters; this enrichment is more pronounced than for DHSs (approximately 40%), suggesting a presence of H3K9ac at promoters in the absence of DHSs. The number of intergenic H3K9ac-enriched regions, excluding promoters, was 6511 in total; 3115 in V2-IST, 6213 in husk and 2668 shared between both tissues (Table 1B).

The overall H3K9ac-enriched regions occupy 2% and 7% of the uniquely mappable genome for V2-IST and husk, respectively (Fig. 3i, k). The fraction in husk is larger than in V2-IST because there were 1.5-fold more H3K9ac-enriched regions in husk and these regions were also longer (Additional file 1: Figure S4A, medians of 603 bp and 1015 bp in V2-IST and husk, respectively). The latter aspect is partly due to merging H3K9ac-enriched regions from three replicates for husk and two for V2-IST. Interestingly, despite the increase in H3K9ac-enriched regions in husk compared to V2-IST, no difference in the distribution of gene expression levels between the two tissues was observed (Additional file 1: Figure S4B). This observation suggests that the number of active genes is similar between the two tissues and independent from the identified number of H3K9ac-enriched regions.

### 46,935 intergenic regions with low DNA methylation are potential enhancer candidates

Low DNA methylation was selected as the third feature to identify enhancers because of its positive correlation with enhancer activity in mammals and plants [29, 36, 55–58]. To count the number of potential enhancers in the B73 maize genome, publicly available BS-seq data obtained from B73 coleoptile shoots were used [35]. Studies in Arabidopsis have revealed that DNA methylation levels in CG (mCG) and CHG (mCHG) contexts (H being A, C or T) are highly stable in different vegetative tissues [59, 60]. Furthermore, locus-specific [36] and genome-wide studies in maize ([61]; RO, MS and NMS, unpublished observations) provided little evidence for changes in mCG or mCHG levels in different vegetative tissues, justifying the use of the coleoptile shoot dataset. We identified regions with 20% or lower DNA methylation in CG and CHG contexts independently, followed by defining LUMRs as regions that were low in both mCG and mCHG. Data on DNA methylation in CHH context (mCHH) were not included in the enhancer prediction step since, compared with the average levels of mCG and mCHG (86% and 74%, respectively), mCHH levels are generally low in maize (2%), like in other plant species [35, 62, 63]. The distribution of LUMRs within the genome was investigated by counting their number in each genomic region (Fig. 3l). The distribution of LUMRs in the uniquely mappable genome revealed an enrichment at genic regions, especially in exons, and at promoters (*p* values < 0.001; permutation test for all genomic categories), but a scarcity at TEs (*p* value = 1; permutation test for TEs); this observation is coherent with the fact that most TEs are highly methylated [35, 64, 65]. Investigation of the LUMR fractions revealed that nearly 50% of the genic regions are lowly methylated, which increases to nearly 60% for promoter regions and exons, while almost all TEs are highly methylated (Fig. 3m). To identify potential intergenic enhancer candidates, we focused on intergenic LUMRs, excluding promoters. We identified 46,935 intergenic LUMRs as potential enhancer candidate regions.

### Integration of features for enhancer candidate prediction

To predict enhancer candidates, we integrated the DHS, H3K9ac and LUMR datasets discussed above. First, we calculated how many LUMRs and DHSs, or LUMRs and H3K9ac-enriched regions, overlapped by at least 1 bp with each other. The overlap between the chromatin features was investigated in both tissues and revealed that more than 97% and 99% of the intergenic DHSs and H3K9ac-enriched regions, respectively, overlapped with LUMRs (Table 1). DHSs are generally shorter than LUMRs (Additional file 1: Figure S4A; median of 484 and 452 bp for V2-IST and husk, versus 834 bp, respectively). While most DHSs or H3K9ac-enriched regions co-localised within LUMRs, only about 20% of the total DHSs and H3K9ac overlapped with each other (Table 1).

Active enhancers are expected to be indicated by a coincidence of chromatin accessibility, H3K9ac enrichment and low DNA methylation [29, 36]. We therefore filtered LUMRs based on the presence or absence of DHSs and H3K9ac-enriched regions and defined LUMRs overlapping with both DHSs and H3K9ac-enriched regions as active enhancer candidates (Fig. 2). Respectively, 398 and 1320 candidates in V2-IST and in husk were identified, of which 223 were shared between the tissues, resulting in 1495 enhancer candidates in total (Additional file 2: Dataset 1 and Additional file 3: Dataset 2). A total of 256 V2-IST and 775 husk candidates were located more than 5 kb away; and 208 V2-IST and 623 husk candidates were located more than 10 kb away from their closest flanking genes. In V2-IST and husk tissue, the median distances between the candidates and their closest genes were 11.4 kb and 8.4 kb, while the largest distances were 438 kb (Zm00001d004626) and 498 kb (Zm00001d030489), respectively. Intersection of our candidates with a published dataset of sequence comparisons between rice and maize genomes indicated that 41 (10%) V2-IST and 241 (18%) husk candidates contained conserved non-coding sequences (CNSs). The overlap between enhancer candidates and CNSs is higher than expected for randomized features ([66], *p* value < 0.001; permutation test).

### Enhancer candidates and transposable elements

Interestingly, 133 (33%) V2-IST and 370 (28%) husk candidates overlapped by at least 1 bp with TEs (Table 2). In most cases, enhancer candidates intersecting with TEs (TE-enhancer) overlapped more than 80% of their length or were entirely located within TEs. The number of TE-enhancers is the highest for long terminal repeat (LTR) retrotransposons, followed by helitrons and terminal inverted repeat (TIR) TEs, consistent with the fraction of the genome the three orders of TEs contribute to the TE space of the maize genome [39]. This TE space is calculated taking the average length for TEs and their number into account (136,000 LTRs with an average length of 9282 bp, 21,000 helitrons with an average length of 3605 bp and 14,000 TIRs with an average length of 621 bp). A small number of TIR elements (seven) are embedded entirely within enhancer candidates, possibly representing rare cases where the insertion of a small TE into open chromatin does not disrupt enhancer function. Indeed, these seven TIRs are in the range of 83–199 bp; one overlaps with an H3K9ac peak, six do not overlap with either a DHS or H3K9ac peak; all are enriched in mCHH (Additional file 1: Figure S5A and B). To further assess the potential of TEs to create enhancers, for the remaining analyses we focused on the subset of TEs that contained at least 80% of an enhancer (Table 2).

**Table 2:** Summary of overlap between enhancer candidates and TEs

		TE within enhancer candidate			Enhancer candidate entirely within TE			Enhancer candidate 80% within TE			Any overlap		
	All	LTR	TIR	Helitron	LTR	TIR	Helitron	LTR	TIR	Helitron	LTR	TIR	Helitron
V2-IST	398	0	1 (0.2%)	0	83 (20.9%)	1 (0.2%)	10 (2.5%)	90 (22.6%)	1 (0.2%)	12 (3%)	110 (27.6%)	10 (2.5%)	17 (4.3%)
Husk	1320	0	7 (0.5%)	1 (0.1%)	202 (15.3%)	9 (0.7%)	56 (4.2%)	212 (16.1%)	9 (0.7%)	62 (4.7%)	261 (19.8%)	28 (2.1%)	88 (6.7%)

Number of enhancer candidates overlapping fully, by 80% of their length, or at least 1 bp with the TEs indicated. Percentages (in parenthesis) indicate the percent of intergenic enhancer candidates within each category. *LTR* long terminal repeats, *TIR* terminal inverted repeats. ‘All’ indicates all enhancer candidates

The average distance between TEs and their closest genes did not vary between all TEs and TEs containing enhancer candidates (mean distance of 40.4 kb and 42.5 kb, respectively; Additional file 1: Figure S6A and B). The TEs that contain candidates tend to be longer than other TEs. To assess if enhancer candidates are likely to overlap with promoters that create functional transcripts for the TEs, we examined the distribution of the candidates within TEs. They were distributed randomly within the TEs, while functional TE promoters are expected to be located at the TE ends, indicating most candidates within TEs are unlikely to be located at the functional promoter site of TEs (Additional file 1: Figure S6C).

We explored the possibility that certain TE families could be a source of enhancers throughout the genome by looking for examples in which multiple members of the same TE family contained enhancer candidates (Additional file 4: Dataset 3). In most cases, only a single member of a TE family overlapped with enhancer candidates, with the exception of some very large TE families. Enrichment of TE families at enhancer candidates was tested by assuming a binomial distribution and applying Bonferroni correction for multiple testing. Only three TE families showed significant enrichment for enhancer candidates (RLG00010, RLG00357, RLG01570; annotations are available from Gramene [67] and the TE classifications from the Maize TE database [http://maizetedb.org]). The LTR Gypsy family RLG00010 was most significantly enriched (*p* value < 0.001), overlapping with seven V2-IST and 23 husk enhancer candidates. This represents a significant fraction of all TE-enhancers in the two tissues (7% and 8.6% for V2-IST and husk, respectively). The RLG00010 family was selected for further analysis.

The same trends were observed for RLG00010 members overlapping with enhancer candidates as for all TEs: a similar distribution of distances of TEs to their closest flanking gene (Additional file 1: Figure S6B and D), and a longer average length for TEs overlapping with candidates (10,895 bp compared with 8517 bp; Additional file 1: Figure S6A and E). Typical examples of RLG00010 TEs overlapping with enhancer candidates are shown in Additional file 1: Figure S5C. To examine if RLG00010 family members overlapping with enhancer candidates were enriched for specific consensus sequences relative to other family members, several de novo motif analysis tools were used [68–71]. When comparing the results from different algorithms, the GGCCCA motif stood out as recurring (found by MEME with *p* value < 0.0039, DREME with *p* value < 0.043, RSAT Plants with E-value of 2.9e^–7^). This motif, also named site II motif, has been discovered in promoter regions of various genes that are highly expressed, for example ribosomal and DEAD-box RNA helicase genes [72–74]. TCP and ASR5 transcription factors are examples of proteins shown to bind the GGCCCA motif [75, 76]. Scanning for the motif using FIMO [77] revealed that most enhancer candidates contained the GGCCCA motif irrespective of an overlap with the RLG00010 family (Additional file 1: Table S4). In fact, compared with random intergenic sequences, enhancer candidates showed an about twofold enrichment for the motif (*p* < 0.001). In contrast, the motif was not enriched in the RLG00010 family as such irrespective of their association with candidates.

### Characterisation of enhancer candidates

**Fig. 5:**
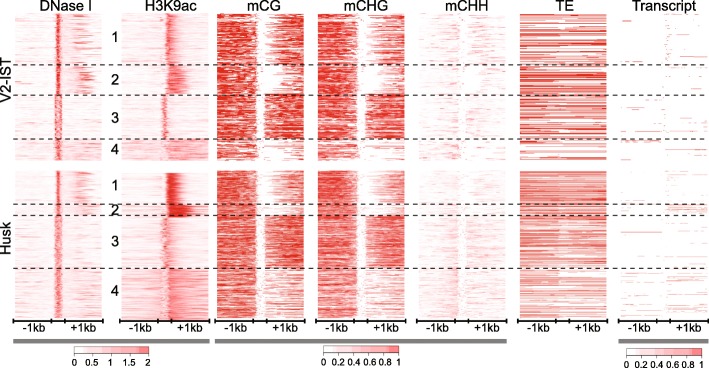
*Heatmaps* of chromatin, DNA and transcript features at enhancer candidates. DNase I hypersensitivity, H3K9ac enrichment, mCG, mCHG and mCHH levels, presence of TEs and transcript levels at and around (±1 kb) DHSs in enhancer candidates. DHSs were scaled to equal size. The *colour scales* are in RPM for DNase I hypersensitivity, H3K9ac enrichment and transcript levels, and in methylation frequency (0–1) for DNA methylation. For TE sequences, *red* and *white* show the presence or absence of TEs, respectively. DHSs were clustered based on H3K9ac enrichment using a k-means (k = 4) clustering algorithm. The categories identified were numbered from 1 to 4 from the top to the bottom. All the DHSs were oriented based on H3K9ac enrichment intensity values 300 bp away from the DHS boundaries; the side with higher H3K9ac enrichment was defined as 3' end

**Fig. 6:**
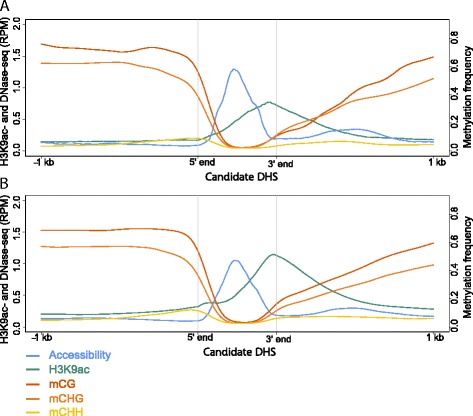
Average profiles of the enhancer candidates in (**a**) V2-IST and (**b**) husk. Average signal intensities of DNase I hypersensitivity, H3K9ac enrichment in RPM and DNA methylation levels in methylation frequency at DHSs and their 1-kb flanking regions. DHSs were scaled to equal size. Prior to calculation of the average, all the DHSs were oriented based on H3K9ac enrichment intensity values 300 bp away from the DHS boundaries; the sides with higher H3K9ac enrichment were defined as 3' end. The profiles show a clear preferential enrichment of H3K9ac 3’ of the DHSs and high levels of DNA methylation (CG and CHG context) around the DHSs and H3K9ac-enriched regions. The level of mCHH is low throughout the regions with a slight increase at the 5’ side of DHSs

**Fig. 7:**
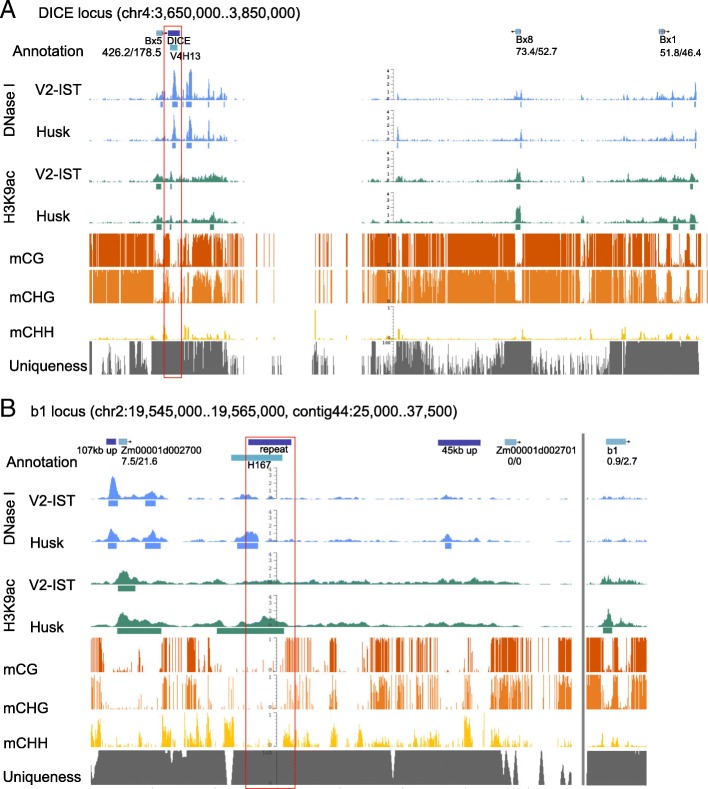
Example of data on (**a**) DICE and (**b**) *b1* repeat enhancer. From the *top*: AGPv4 annotation and candidate annotation from our prediction (*V* V2-IST, *H* husk candidate), DNase I hypersensitivity and H3K9ac enrichment signal (all replicates pooled) and peak position (indicated as *blue* and *green bars*, respectively) in V2-IST and in husk tissue, mCG, mCHG and mCHH levels and unique mappability in percentage. The numbers under gene names indicate relative gene expression levels (V2-IST/husk). Although the *b1* locus is on chromosome 2, in the current version of the AGPv4 assembly, the *b1* gene is located in contig 44 (B, on the *right* of the *grey vertical line*). The *dark blue bars* in the gene annotation tracks indicate previously annotated known enhancers and putative *cis-*regulatory elements. The *vertical red boxes* indicate enhancer candidates identified in this study. Peaks at those tracks might not be present in each replicate, affecting enhancer candidate prediction

In humans, enhancers generally show a bi-directional pattern of DNA, chromatin and transcript features. Histone modifications such as H3K27ac, as well as eRNA transcription, are located at both sides relative to single DHS peaks [4]. We set out to analyse whether DNA and chromatin features at our candidate enhancers displayed directionality. The read coverages for DNase-seq, H3K9ac ChIP-seq and DNA methylation in all three contexts were extracted for each DHS located in enhancer candidates and their 1-kb upstream and downstream flanking regions (431 candidates in V2-IST and 1,437 in husk) (Fig. 5). Note that the number of DHSs was higher than that of enhancer candidates because multiple DHSs could be located in one candidate. The averages of the read coverages are presented in Fig. 6. Empirical observations indicated that H3K9ac was often enriched at only one side of DHSs (see e.g. Fig. 7 and Additional file 1: Figure S7). Therefore, the orientation of DHSs was defined based on H3K9ac enrichment levels 300 bp from DHSs, the sides with the higher H3K9ac enrichment value, if present, being defined as 3' end. The observed asymmetry was further validated by plotting the H3K9ac enrichment values from both sides of the DHSs with and without the previously defined orientations for all DHSs (Additional file 1: Figure S8). For DHSs showing H3K9ac enrichment at either side of at least 0.5 RPM, 241 out of 431 in V-IST and 841 out 1437 in husk showed asymmetric H3K9ac enrichment as indicated by an at least twofold change in H3K9ac enrichment between the two flanking regions.

The enhancer candidates were clustered into four categories based on H3K9ac enrichment patterns using the k-means clustering algorithm and the categories were numbered according to their appearance in the heatmaps (Fig. 5). For each category, average patterns were determined (Additional file 1: Figure S9). Heatmaps and profiles showed that H3K9ac can be primarily enriched on one side of the DHSs (category 1 and 2), within DHSs (category 3) or present at both sides but clearly enriched at one of them (category 4) (Fig. 5 and Additional file 1: Figure S9).

Comparing DNase-seq or H3K9ac ChIP-seq read coverages with the distribution of mCG and mCHG levels, but also the average profiles, indicated that high chromatin accessibility and H3K9ac enrichment levels were exclusive with high DNA methylation levels (Figs. 5 and 6 and Additional file 1: Figure S9). The average profiles show a plateau and steep decline of mCG and mCHG at the 5’ side of DHSs (Fig. 6). In categories 1, 2 and 4, at the 3' side of enhancer candidates, mCG and mCHG levels increased gradually (Fig. 6, Additional file 1: Figure S9). These patterns indicate a sharp transition in DNA methylation level at the 5’ DHS boundaries and a more gradual transition at the H3K9ac boundaries. However, a sharp transition at the 5’ ends of candidates may be masked in the average profile by the varying size of the H3K9ac-enriched regions. In line with this, the profile of category 3 candidates, having H3K9ac at the DHSs itself, showed sharp boundaries at both sides of the candidates. Levels of mCHH were lower than mCG and mCHG levels, as expected [35]. In line with earlier studies [61, 62], mCHH marked boundaries between lowly and highly DNA methylated regions as shown by the relatively high level of mCHH, represented by a small mCHH peak in the average profiles, at the 5’ boundaries of the DHSs (Figs. 5 and 6 and Additional file 1: Figure S9).

Additional heatmaps and profiles were created to illustrate the locations of TEs and transcripts for the four categories. The heatmaps suggest that TEs covered all selected regions, showing a slight depletion across DHSs but no apparent pattern across other features (Fig. 5). In animal models, enhancers are characterised by bi-directional transcription and the transcribed regions are, among others, enriched with H3K27ac [4]. In our data, transcript levels were generally low at candidates except for a few showing transcripts within and/or outside of their DHS (Fig. 5), making the detection of bi-directional transcription very challenging. In addition to this absence of detectable levels of bi-directional transcription, the clear asymmetric H3K9ac distribution at a majority of maize enhancer candidates suggested that the candidates have more resemblance to TSSs than animal enhancers do [4].

### Profiles of DNA and chromatin features at enhancer candidates and TSSs are similar

To rule out the possibility that our enhancer candidates were actually TSSs of unannotated genes, we compared the patterns of their DNA, chromatin features and transcript features with those observed at annotated TSSs by randomly selecting 431 and 1437 DHSs located at TSSs for V2-IST and husk, respectively (Additional file 1: Figure S10). The selected regions were oriented according to the 5’ to 3’ orientation of flanking genes and analysed using the k-means clustering algorithm (k = 3). In general, the heatmaps and average profiles of DHSs at TSSs displayed a strong DNA methylation signal at the 5’ ends of DHSs and an enrichment in H3K9ac and an accumulation of transcripts at the 3' ends of DHSs (Additional file 1: Figure S10 and S11). The heatmaps and the average plots of TSSs and enhancer candidates revealed similar patterns of chromatin accessibility and H3K9ac, but they differed in transcript levels (higher at annotated TSSs) and distribution of mCG and mCHG (high on both sides for candidates, while restricted to the 5’ side for annotated TSSs) (Figs. 5 and 6, Additional file 1: Figures S10 and S11). The median transcript level at the enhancer candidates was 6.6 times lower than that at coding sequences in V2-IST; the fold change could not be calculated for husk because the candidate expression levels had a median of 0 RPKM (Additional file 1: Figure S12). One category (category 3), showed transcriptional activity and H3K9ac enrichment on both sides (Additional file 1: Figure S10). The DHSs in this category were either flanked by two oppositely orientated and closely spaced genes or by alternative TSSs located in upstream regions.

H3K4me3 histone modification was previously described for distinguishing TSSs from enhancers [21, 78–80]. Analysis of published ChIP-seq data for H3K4me3 in maize third seedling leaf [61] indicated that 24% and 11% of the V2-IST and husk enhancer candidates, respectively, overlapped with H3K4me3 enriched regions (Additional file 1: Figure S13), which could hint at unannotated TSSs. The observed H3K4me3 enrichment at enhancer candidates was, however, on average weaker than at TSSs (Additional file 1: Figure S13), suggesting H3K4me3 may also differentiate TSSs and enhancers in maize. In addition, the H3K4me3 enrichment pattern did not entirely reflect the H3K9ac enrichment pattern at TSSs but was rather slightly shifted downstream of the H3K9ac peaks. Such a pattern has not been reported in humans [79] and was not observed in a previous study in rice [21].

In summary, despite a shared polarity with respect to flanking H3K9ac enrichment, the profiles of enhancer candidates differ from those at TSSs by the levels of transcript accumulation, DNA methylation and H3K4me3.

### Ranking and selecting a list of tissue-specific enhancer candidates

**Fig. 8:**
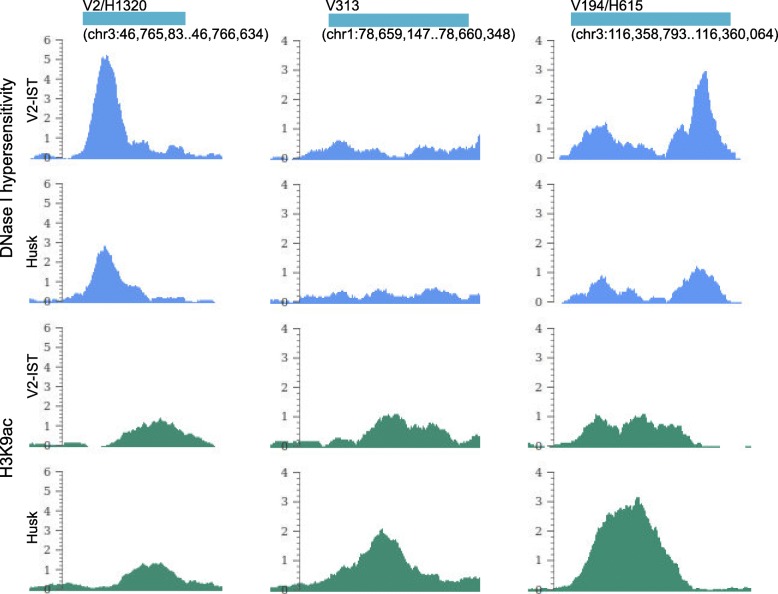
Examples of candidate rankings. From the *top*: identified candidate region with its ID (*V* V2-IST, *H* husk candidate) and coordinates, DNase I hypersensitivity and H3K9ac enrichment signal intensities in V2-IST and husk tissues. In these examples, the DNase I hypersensitivity and H3K9ac enrichment signal differences do not positively correlate to each other as assumed

To facilitate linking enhancer candidates to putative target genes, we set out to determine the degree of tissue-specificity of our enhancer candidates by ranking the 398 V2-IST and 1320 husk candidates based on the assumption that the levels of both DNase I hypersensitivity and H3K9ac enrichment are positively correlated with enhancer activity. The enhancer candidates were independently ranked based on the largest differences between the two tissues for DNase I hypersensitivity and H3K9aclevels. The strongest tissue-specific candidates were assumed to exhibit large differences in both DNase I hypersensitivity and H3K9ac enrichment; therefore, the independent rankings for both features were summed for every candidate and the candidates were re-ranked (Additional file 2: Dataset 1 and Additional file 3: Dataset 2, column overall_rank). The ranking numbers were combined with a V for V2-IST or an H for husk as candidate IDs; the lower the number, the more tissue-specific the candidate. However, the rankings for DNase I hypersensitivity and H3K9ac enrichment did not correlate with each other (Additional file 2: Dataset 1 and Additional file 3: Dataset 2, column DNase_rank and H3K9ac_rank; shared candidates were ranked in both tissues). For example, the candidate ranked to the second place (candidate V2, Fig. 8) for V2-IST showed a large difference in DNase I hypersensitivity signal between V2-IST and husk tissue as expected, while the H3K9ac enrichment stayed almost the same for both tissues. The 313th candidate in V2-IST (candidate V313), on the other hand, is characterised by a large difference in H3K9ac enrichment but not in DNase I hypersensitivity. The 194th candidate in V2-IST (candidate V194) showed a large difference between the tissues for both DNase I and H3K9ac enrichment signals but in an opposite direction. The lack of correlation between the ranks obtained from both chromatin features indicated that determining tissue-specificity using this combination of features does not work properly. Experimental examinations of a number of candidates will be necessary to determine the best feature (combination) to predict tissue-specificity. For now, enhancer candidates identified in only one of the two tissues were defined as tissue-specific and the shared candidates between tissues as putative shared enhancers. With this definition, a total of 1495 candidates were classified into 175 V2-IST-specific, 1097 husk-specific and 223 shared candidates (Additional file 5: Dataset 4).

### Predicting putative target genes of enhancer candidates based on expression levels of closest genes

Lastly, we examined if our candidates could be linked to putative target genes. Multiple approaches have been reported using data on chromatin accessibility, transcript levels and/or histone modification patterns at both enhancers and genes, across different tissues or developmental time points [4, 51, 81, 82]. We assumed that enhancers regulate the expression of either their adjacent upstream or downstream gene, though it has been observed that other genes can be located between enhancers and their target genes in animals and plants [17, 83–85]. We correlated the defined tissue-specificity of candidate enhancers with the gene expression levels of the nearest flanking genes in both tissues. Only genes showing significant differential expression between V2-IST and husk tissue (Cuffdiff [86]) were considered as targets of tissue-specific enhancer candidates; for shared candidates, flanking genes that are expressed in both tissues were considered as potential target genes. If a flanking gene showed a significant difference in gene expression that matched the enhancer candidate specificity (e.g. higher gene expression in V2-IST for V2-IST candidates), then the candidate and the gene(s) were linked. With this method, 38 (22%) V2-IST-specific, 143 (13%) husk-specific and 101 (45%) shared enhancer candidates were linked to one putative target gene (Additional file 5: Dataset 4). We also identified 13 (2%) V2-IST-specific, 182 (17%) husk-specific and 103 (46%) shared candidates in which both flanking genes showed expression levels matching the features of the candidates. The other candidates could not be linked to a gene because either none of the flanking genes had a significant expression level difference in the expected direction for tissue-specific candidates (124 [71%] in V2-IST, 772 [70%] in husk) or, in case of shared enhancer candidates, neither of the flanking genes were expressed in one of the tissues (19 [9%] candidates).

### Identification of three known enhancers in maize

In maize, five well-characterised and putative enhancers were reported, namely the *b1* hepta-repeat, the enhancers of *tb1*, *p1*, and the putative enhancers DICE and *Vgt1* that regulate the expression of the genes *bx1* and *ZmRAP2.7*, respectively [11, 13–15, 23, 85, 87]. In our screen, we identified the confirmed and putative enhancers of *b1, tb1* and *bx1* (Fig. 7 and Additional file 1: Figure S7), although these enhancers were mostly identified and characterised in maize lines other than B73, which could have affected their functionality. For example, the *b1* hepta-repeat enhancer has been identified for the *B-I* epiallele and consists of seven copies of an 853-bp sequence in tandem, while B73 only carries a single copy of this sequence (90% identity with consensus repeat sequence) [12]. In our dataset, *b1* showed differential expression in the same direction as observed in the line the *b1* repeat enhancer was discovered [23], already indicating there is some degree of conservation in the regulatory region. The *tb1*enhancer was identified in the inbred line W22 [13, 14] and DICE was shown to be required for high *bx1* expression in Mo17 [85]. The enhancer candidates for *b1* and DICE were not linked with *b1* and *bx1*, respectively, because their known target genes were not the closest flanking gene. We identified neither the *p1* enhancer nor *Vgt1.* In the case of the *p1* locus, high repetitiveness of the region rendered the enhancer unmappable. For *Vgt1*, a clear DHS was present but H3K9ac-enrichment was not detected within the overlapping LUMR.

Four H3K9ac-enriched enhancer candidate regions identified by ChIP-seq, candidate H108, the *b1* and *tb1* enhancer and DICE, were selected for validation with ChIP-quantitative polymerase chain reaction (qPCR). For each region, primer pairs were designed to amplify sequences located at the summit of the peak of the ChIP-seq H3K9ac-enriched region (P), its slope (S) and outside of the peak (O; no enrichment by ChIP-seq) (Additional file 1: Figure S14). Results confirmed the presence and absence of H3K9ac enrichment at the identified candidate regions and their flanking regions, respectively. The differential H3K9ac enrichment observed for candidate H108 and the *b1* enhancer fits their expected husk tissue-specificity based on the ranking. DICE had a high and low ranking in V2-IST and husk, respectively. In accordance, DICE showed higher H3K9ac enrichment levels in V2-IST than in husk. The *tb1* enhancer showed H3K9ac enrichment in both V2-IST and husk. This is in accordance with what is observed for the pooled ChIP-seq data (Additional file 1: Figure S14C). Due to our stringent criteria, the *tb1* enhancer was only called as a candidate in husk.

To examine if H3K4me1 is indeed not enriched at enhancers as suggested by the results depicted in Fig. 1, enrichment for H3K4me1 was determined for the same regions as for H3K9ac enrichment (Additional file 1: Figure S14). Except for the enhancer of *tb1*, none of the analysed regions showed a clear H3K4me1 enrichment, confirming our previous observation and supporting the idea that H3K4me1 does not generally mark plant enhancers.

## Discussion

The combination of DNase-seq, H3K9ac ChIP-seq and BS-seq data allowed us to identify approximately 400 and 1300 enhancer candidates in V2-IST and husk tissue, respectively, and about 1500 unique enhancer candidates in total. Interestingly, our enhancer candidates displayed an asymmetric enrichment of H3K9ac at DHSs, which differs from the histone acetylation enrichment at both sides of DHSs observed in animals [4, 27, 29]. Target genes were predicted for 255 V2-IST and 529 husk candidates. Importantly, our method successfully predicted three confirmed or putative enhancers in the maize genome, enhancers for the *b1* (candidate H167) and *tb1* (candidate H1233) genes and the DICE enhancer (candidates V4 and H1318).

We investigated the enrichment of three histone modifications at the enhancer of *b1*: H3K27ac, H3K9ac and H3K4me1, and showed that both H3K27ac and H3K9ac were enriched at the hepta-repeat enhancer of *b1* in the active, but not the inactive, state*.* These results are in accordance with previous studies in animals, but also in plants [20, 28, 30, 36, 37, 88]. In contrast, H3K4me1, which was shown to be enriched at animal enhancers regardless of their activity [27, 79], was not enriched at the *b1* hepta-repeat enhancer, but also not at DICE and candidate H108 (Additional file 1: Figure S14), while it was present at relatively high levels at transcribed regions of *b1* and *actin1* (Fig. 1). This distribution at enhancers may be typical for plants as it is supported by previous observations in Arabidopsis in which H3K4me1 was almost exclusively enriched in genic regions [89].

Regions with low DNA methylation overlap with DHSs and both were previously used to predict enhancers [29, 90]. In our study, more than 97% of DHSs and more than 99% of H3K9ac-enriched regions overlapped with LUMRs; enhancer candidates were identified by taking the overlap between LUMRs, DHSs and H3K9ac-enriched regions, resulting in about 1500 unique enhancer candidates. Many more intergenic LUMRs were identified (about 47,000) and 26% of these carried only one, while 71% carried none of the other required chromatin features. We hypothesize that these remaining LUMRs represent enhancers in tissues other than the ones used in our study. This could in part explain the relatively low number of identified candidates compared with studies in animals in which a large number of developmental stages, cell types and/or tissues were used [4, 51, 83]. In Arabidopsis, more than 10,000 intergenic enhancer candidates were predicted using only two different tissues [20], as we did. However, the authors based their prediction solely on chromatin accessibility. Based on chromatin accessibility data only, we would predict about 9000 candidate enhancers. Instead, we used a more stringent approach to identify active enhancers.

Ten percent and 18% of V2-IST and husk candidates contained previously published CNSs between maize and rice [66], suggesting these candidate sequences and functions may be conserved across species. The rest of the candidates might be maize-specific or rapidly diverging [91], explaining the lack of sequence conservation. About 30% of the enhancer candidates in both tissues overlapped by at least 1 bp with TEs (33% in V2-IST and 28% in husk) and in most cases TEs covered the entire enhancer candidate region. This raises questions regarding the origin of the regulatory potential of those enhancer candidates. Indeed, TEs have been reported as an important source of *cis*-regulatory elements because TEs have evolved to mimic the regulatory sequences of the host to hijack its transcriptional machinery [14, 38, 92–94]. Three LTR Gypsy families were significantly enriched for enhancer candidates. Motif analysis of the enhancer candidates overlapping with the most enriched TE family, RLG00010, identified the GGCCCA motif, which is discovered in *cis*-regulatory elements of genes with diverse functions [72, 73, 75, 76]. Compared with random intergenic sequences, this motif was not only enriched in the RLG00010 enhancer candidates, but also in all other candidates. This suggests that GGCCCA is a general motif associated with enhancer function.

Although we identified three previously discovered putative or confirmed enhancers in maize, two others, *Vgt1* and the enhancer of *p1*, were not detected. This can be explained by several factors: (1) enhancer sequences can be located in repetitive regions, which are not uniquely mappable and therefore excluded from our analysis (true for the *p1* enhancer); (2) enhancers may not always require the stringent criteria used to define enhancer candidates in this study (could be true for *Vgt1*, which featured an LUMR and DHS but no H3K9ac-enriched region); (3) enhancers may not be active in V2-IST or husk tissue and therefore undetected; and (4) enhancers may only be present in other lines than B73.

We identified about three times more enhancer candidates in husk tissue than in V2-IST (398 versus 1320), which is possibly due to a larger number of H3K9ac-enriched sequences in all genomic regions in husk compared to V2-IST (Fig. 3h and j). There was, however, no difference in the distributions of gene expression levels between the two tissues (Additional file 1: Figure S4B), indicating that the number of genes expressed at particular levels is similar in V2-IST and husk and that the larger number of H3K9ac-enriched sequences is therefore not due to a higher number of genes being expressed in husk. The differences in the number of H3K9ac-enriched regions were substantial, even when considering possible technical bias introduced during the analysis. This observation highlights that the H3K9ac enrichment pattern changes between tissues and/or developmental stages, irrespective of the overall distribution of expression levels. The reasons for this change are currently unknown.

The heatmaps and average profiles of the chromatin and DNA features at the candidates revealed that H3K9ac was preferentially enriched on one side of the DHSs (Figs. 5 and 6). This observation was unexpected considering earlier studies in animals describing histone acetylation (H3K27ac), but also methylation (e.g. H3K4me1) enrichment on both sides of DHSs at enhancers [4, 27, 29]. Symmetrical enrichment of histone modifications at animal enhancers has been associated with bi-directional transcription at enhancers [4]. Given the relative low coverage of our RNA-seq data at enhancer candidates, we were not able to assess whether eRNAs were produced bi- or uni-directionally. eRNAs are indeed known to be transcribed at a low level and in addition sensitive to degradation, making them difficult to detect with a technique such as RNA-seq [4, 95]. The analysis of nascent transcript data (GRO-seq) for maize and Arabidopsis suggests the absence of transcription at plant enhancers [96], further supporting the possible differences between plant and animal enhancers. A method like CAGE-seq could be used to further investigate the transcription of enhancers in plants.

Elevated levels of mCHH were detected 5’ of the DHSs at enhancer candidates. mCHH islands have been observed to flank genic regions in maize, but also low DNA methylated intergenic CNSs [61, 65]. The findings of Li et al. [61] showed that mCHH islands may act as boundaries between euchromatin and heterochromatin, preventing activation of TEs by nearby transcriptionally active genes. A similar function is likely at enhancers.

Comparison between the chromatin and DNA methylation profiles at enhancer candidates and TSSs revealed the presence of similar features, including chromatin accessibility, asymmetric H3K9ac enrichment and low DNA methylation. On average, the TSSs show a higher level of transcript accumulation, a lower level of DNA methylation 3’ of TSSs and a higher level of H3K4me3 than enhancer candidates (Fig. 5, Additional file 1: Figure S9, Figure S11 and Figure S13). The difference in transcript levels and H3K4me3 enrichment between enhancers and TSSs has been observed by others [4, 79].

For each enhancer candidate, a target gene was predicted following expression and proximity criteria. Our prediction method assumed that target genes were either the adjacent upstream or downstream gene and that target genes of tissue-specific enhancer candidates would be upregulated in the tissue in which the enhancer candidates were detected. Using our stringent criteria, 580 candidates were linked to genes, including *tb1*. In Drosophila, about 20% of the enhancers were predicted to control genes that were not directly adjacent to the enhancers [83] and a recent prediction in human and mice estimated that 69% of the enhancers contact genes that are not directly consecutive [82]. Whether this proportion is similar in maize remains to be determined, but examples of such enhancers have been reported, for example DICE, the putative enhancer of *bx1* [85]. In addition, our approach disregarded the possibility that enhancer candidates would act as transcriptional repressors [97]. Future studies in maize are required to more precisely identify and validate the target genes of the enhancer candidates discovered.

## Conclusions

This study provides a genome-wide glance at transcriptional enhancer candidates in maize by comparing DNA and chromatin features in two maize tissues and by providing details on some of their characteristics. The study identified about 1500 enhancer candidates that were characterised by increased chromatin accessibility, low DNA methylation levels and asymmetric enrichment of H3K9ac. Three identified candidates were putative or confirmed enhancers (*b1*, *tb1* and *bx1* enhancers). In contrast to animals, plant enhancer candidates show asymmetric chromatin features. Validation of enhancer candidates remains to be achieved. Future improvements in predicting enhancer candidates are expected from the investigation of more histone modifications as well as TF binding sites, the integration of genome-wide chromosomal interaction data and a direct functional analysis of candidates, e.g. by targeted genome editing. A better understanding of the regulatory code in maize not only helps to better compare transcription regulation in highly complex genomes of different kingdoms but promises new targets for informed breeding in this important crop. Our data provide a framework for the maize community to characterise the regulation of genes of interest.

## Methods

### Experimental methods

#### Plant stocks and material

The seed stock of the maize B73 inbred line used in this study was obtained from J. Gardiner (University of Arizona, Tucson, AZ, USA) in 2013. It was obtained from the North Central Regional Plant Introduction Station in Ames, IA, USA (order no.: 169545, accession: PI550473, lot: 94ncai02). It is from the same accession (PI 550473) that was used for the maize B73 genome sequencing project [41], but a different lot number because it was requested several years later. The *B-I* plant stock used in this study (W23) was obtained from V.L. Chandler (University of Arizona, Tucson, AZ, USA). Maize plants were grown in the greenhouse at two different locations: The Max Planck Institute for Plant Breeding Research in Cologne (MPIPZ) and the University of Amsterdam (UvA). At the MPIPZ, maize plants were grown for DNase-seq and RNA-seq. At the UvA, maize plants were grown for H3K9ac ChIP-seq and RNA-seq. At both locations plants were grown in soil under 16-h/8-h light/dark cycles at an average temperature of 23 °C. The plants were harvested at the V2 stage (two collars visible; V2-IST), V5 stage (five collars visible; V5-IST) or when the silks started emerging from the husks. The two tissues used for the RNA-seq, DNase-seq and ChIP-seq experiments were the inner stem tissue of V2 seedlings, which is composed of the seedling stem with the outer leaves and all exposed leaf blades removed, and the soft inner husk leaves surrounding the ear; the tough outer husk leaves were discarded (Additional file 1: Figure S1).

#### RNA-seq

RNA for RNA-seq experiments was isolated at both locations. To be able to examine reproducibility and comparability, per tissue, three biological replicates were analysed, each consisting of pooled material from three plants. The inner husk leaves and inner stem tissue of V2 seedlings were flash frozen in liquid nitrogen 9–11 h after dawn. After grinding in liquid N_2_, 100 mg material was used for RNA extraction with TRIzol (ThermoScientific) following the manufacturer’s instructions except that the top aqueous phase was transferred to a new tube, 500 μL of isopropanol were added, followed by mixing and incubation for 10 min at RT. The entire sample was transferred in two steps to an RNeasy MINI spin column (Qiagen RNeasy kit) and centrifuged for 15 s at 8000 × g. The flow-through was discarded and 700 μL of the Qiagen RW1 buffer was added. Two washing steps were performed using 500 μL of the Qiagen RPE buffer. RNA was eluted in 50 μL RNase-free water and the concentration was assessed spectrophotometrically (Nanodrop, ThermoScientific). Next, RNA samples were diluted to a concentration of 200 ng/μL and treated with DNase I (DNA-free kit, Ambion) according to the manufacturer’s instructions. Samples were then extracted with 1 volume of phenol:chloroform:isoamyl alcohol (25:24:1 v/v) and centrifuged for 5 min at 13,000 × g at 4 °C. The same step was repeated twice. Next, 80% of the aqueous phase volume was transferred into a new tube and precipitated with 1/10th volume of 3 M Sodium Acetate pH 5.6, two volumes of 100% ethanol and 1 μL of glycogen (10 mg/mL), followed by centrifugation at 13,000 × g for 15 min at 4 °C. The pellet was subsequently washed twice with 70% ethanol and finally resuspended in 20 μL of RNase-free water. The concentration was measured spectrophotometrically (Nanodrop, ThermoScientific) and 1 μg of RNA was separated on a 1.2% agarose 1× MOPS (3-N-morpholinol propane sulfonic acid) gel to assess RNA quality. The concentration was adjusted to 400 ng/μL and 500 ng of total RNA was treated with the Ribo-Zero rRNA Removal Kit (Plant Leaf, Epicentre) to specifically remove ribosomal RNAs. RNA-seq libraries were prepared with the NEBNext Ultra™ Directional RNA Library Prep Kit for Illumina sequencing (New England Biolabs). Quality and quantity were assessed at all steps of the library preparation by capillary electrophoresis (Agilent Bioanalyser and Agilent Tapestation). Sequencing was performed with TruSeq v3 chemistry on a HiSeq2500. Approximately 15–20 million of 100-bp single-end reads were obtained for each library.

#### DNase-seq

##### Nuclei preparation

For each inner stem tissue sample (V2 stage) and inner husk leaf sample, nuclei were extracted from 12 V2 stage maize seedlings and three husks according to the protocol of Steinmüller and Appel [98]. For each tissue, two biological replicate samples were used. Briefly, tissue was ground in liquid nitrogen, 5 g were transferred into an ice-cold 50 mL centrifuge tube, 25 mL of cold nuclei isolation buffer (20 mM Tris-HCl pH8, 250 mM sucrose, 5 mM MgCl2, 5 mM KCl, 40% glycerol, 0.25% Triton X-100, 0.5 mM EGTA pH 8, 5 mM EDTA pH8, 0.1 mM PMSF, 0.1% 2-mercaptoethanol, 1:1000 Proteinase Inhibitor Cocktail (Sigma)) were added and the tube was flicked until the powder was in suspension. The tube was rotated at low speed at 4 °C until the sample was completely thawed (about 30 min). The tissue suspension was filtered through successive layers of 60 μm and 20 μm nylon mesh (Nylon Net Filters, Millipore) into an ice-cold 50 mL centrifugation tube and centrifuged at 6000 × g for 15 min at 4 °C. The supernatant was discarded and the pellet resuspended in 15 mL of ice-cold nuclei isolation buffer using a 1 mL cutoff pipette tip, followed by centrifugation at 6000 × g for 12 min at 4 °C. The pellet was resuspended in 10 mL of ice-cold nuclei isolation buffer and centrifuged at the same conditions again, followed by resuspending the pellet in 1 mL of ice-cold nuclei storage buffer (20% glycerol, 20 mM Tris pH 7.5, 5 mM MgCl2, 1 mM DTT). To check the quality and abundance of the nuclei, a 20-μL aliquot was stained with 1 μL DAPI (1 mg/mL) and examined by fluorescent microscopy. The nuclei suspensions were flash frozen in liquid nitrogen and stored at –80 °C until further use.

##### DNase I digestion

DNase I treatment was adapted from Chandler et al. [99]. Nuclei suspensions were thawed on ice while preparing the solutions for DNase I digestion. One undigested control and four concentrations of DNase I (50, 100, 150 and 200 U/mL) were used (Additional file 1: Figure S15). In total, 2.5 mL of DNase I buffer (50 mM Tris pH8, 250 mM sucrose, 100 mM KCl, 0.1 mM CaCl2, 5 mM MgCl2, 50 μg/mL BSA, 0.05 M beta mercaptoethanol) was prepared per sample. The DNase I dilutions were prepared by mixing DNase I (Roche) with DNase I dilution buffer (20 mM Tris pH7.5, 50 mM NaCl, 1 mM DTT, 100 μg/mL BSA, 50% glycerol). A total of 1 mL of nuclei suspension was divided in 5 × 200 μL in 1.5-mL microcentrifuge tubes using cutoff pipette tips. The tubes were centrifuged at 1500 × g for 5 min at 4 °C and the supernatant was discarded. A total of 100 μL of 100 mM EDTA pH 8, followed by 600 μL of phenol/chloroform/isoamylalcohol (25:24:1 v/v), were added to the tube for the undigested control and set aside at room temperature after thorough mixing. The other pellets were resuspended in 475 μL of cold DNase I buffer by rubbing the tubes against a plastic tube rack and letting them incubate for 3 min at 25 °C. In total, 25 μL of each of the DNase I dilutions were added to the respective tubes with nuclei suspensions and incubated for 10 min at 25 °C. The reaction was stopped by adding 100 μL of 100 mM EDTA pH 8, mixing and adding 600 μL of phenol/chloroform/isoamyalcohol. All samples, including the undigested control, were shaken by hand or using a tissue lyser (Qiagen) at 8 Hz for 5 min. A second phenol/chloroform/isoamyalcohol extraction was performed, followed by an RNase A treatment (2 μg/mL final concentration) at 37 °C for 10 min. Totals of 600 μL isopropanol, 50 μL of 7.5 M ammonium acetate and 2 μL of 10 mg/mL glycogen were added followed by centrifugation at 16,000 × g for 30 min at 4 °C. Two 70% ethanol washings were performed and the pellets were finally resuspended in 30 μL 10 mM Tris-HCl pH 8.5. The concentration of nuclei acids was then assessed spectrophotometrically (Nanodrop, ThermoScientific) and the entire sample (30 μL) was mixed with 6 μL Cresol Red loading buffer (1.75 M sucrose (60%), 5 mM cresol red, pH 8) and loaded on an agarose gel (1× TAE buffer, 1.5% agarose, 0.5 μg/mL ethidium bromide). Gel visualisation under ultraviolet light indicated which digestion fulfilled the requirement that the DNA is only partially digested (Additional file 1: Figure S15). In our hands, these were the samples digested with 50 U/mL of DNase I. One should test several concentrations as the digestion efficiency can vary depending on the batch of DNase I enzyme and chromatin concentration. The DNA fractions in the range of 100–300 bp were extracted from the gel using gel purification (NucleoSpin Gel, Macherey Nagel) and the DNA was eluted from the column in 15 μL of 10 mM Tris-HCl pH 8.5. The DNA concentration was measured using Quant-iT PicoGreen (Invitrogen) on a fluorometer (Synergy 4 Hybrid Multi-Mode Microplate Reader, BioTek). A DNA concentration range of 1–3 ng/μL was obtained.

##### Naked DNA control

gDNA was extracted from 100 mg of inner husk tissue derived from three pooled husks using the DNeasy Plant Mini kit (Qiagen) and following the manufacturer’s instructions. A total of 1.7 μg of gDNA was digested with 50 U/mL of DNase I following the same protocol as described for chromatin.

##### Library preparation and sequencing

DNA samples were diluted to 1 ng/μL in a total volume of 10 μL followed by library preparation using the Ovation Ultralow DR Multiplex kit (NuGEN) according to the manufacturer’s protocol. Fifteen cycles of amplification were performed for the naked DNA sample and 16–18 cycles for the chromatin-derived samples. The libraries were sequenced on an Illumina Hi-Seq2500 platform and approximately 20–30 million 100-bp single-end reads were obtained for each library.

#### ChIP-seq and ChIP-qPCR

The ChIP procedure was based on the original protocol from Haring et al. [100] with minor modifications. In short, plant samples (five inner stems from V2 plants or 3 g of inner husk leaves per sample) were fixed with formaldehyde. Chromatin was extracted and sonicated. The soluble fraction was then immunoprecipitated using antibodies against H3K9ac (Abcam, ab10812), H3K27ac (Abcam, ab4729), H3K4me1 (Abcam, ab8895) or rabbit serum (No antibody control, Sigma no. R9133) using protein-A coated magnetic beads (ChIP-seq, Diagenode, kch-802) or protein-A agarose beads (ChIP-qPCR, Sigma-Aldrich). Immunoprecipitated DNA was recovered, decrosslinked and column-purified (Qiagen, 28104). For each ChIP-seq library, three ChIP samples were pooled yielding about 50 ng of DNA prior to adapter ligation and PCR amplification. Adaptor ligation (TrueSeq Universal adapter, Illumina) and PCR amplification were performed for each pooled ChIP sample using the KAPA Hyperprep kit (KAPA, KK8500) as indicated by the manufacturer. The efficiency of the conversion process was assessed by comparing the input ChIP sample to the obtained ChIP-seq library on an Agilent High Sensitivity D1000 ScreenTape System. Efficient conversion corresponds to a visible 100 bp shift in fragment sizes and an unbiased increase in DNA concentration. For all samples, approximately 30 million 100-bp single-end reads were generated on an Illumina HiSeq2500 platform.

For ChIP-qPCR, the column-purified material (4 μL out of 80 μL) was mixed with 2 μL of each primer (10 μM; Additional file 5) and 4 μL of the 5X FIREPol Evagreen qPCR Mix plus (Solis Biodyne) in a total volume of 20 μL and run on an Applied Biosystem 7500 Real Time PCR system (50 °C, 2’; 95 °C, 10’, 45 cycles: 95 °C, 15”; 65 °C, 1’). For each primer pair, a calibration curve was generated using DNA isolated from fixed, sonicated chromatin (100 ng/μL; dilutions 1/64, 1/256 and 1/1024) to test primer efficiency and calculate DNA quantities from ChIP samples. Enrichment is calculated as the mean quantity of the different biological replicates (2–5) and normalized over the quantity at the maize *actin* locus. All PCR primer sequences are listed in Additional file 6: Table S5.

#### Computational analysis

For all the analysis, the B73 maize genome sequence and annotation version 4 (AGPv4) [39] from Ensembl Plants [40] were used as the reference. Data on chromosomes 1 to 10, excluding contigs, were used for all the analysis. For statistical enrichment analysis, permutation tests were performed (n = 1000) [101]; the randomisation of features within the uniquely mappable part of genome was performed using BEDtools [102].

#### RNA-seq

The sequenced reads were trimmed at the both ends based on sequencing quality (Q20) and remaining Illumina adaptor sequences were removed using Trimmomatic [103]. When the remaining read length was less than 35 bps, the read was removed from the analysis. The reads were aligned, allowing one mismatch, to the reference genome using TopHat2 [104] and Bowtie [105]. Transcript assembly and gene expression level calculation for each replicate were performed with a guided reference [40] using the Cufflinks pipeline (Cufflink, Cuffquant and Cuffnorm) [106]. The RPKM values and the significance of the differential expression levels for each gene were calculated taking the variance over the six replicates using Cuffdiff [86]. The RPM coverage in the genome was calculated using BEDtools [102].

#### DNase-seq and ChIP-seq

For DNA-seq data, to assess technical variation, two independent DNase-seq libraries were generated from one biological husk sample and the number of shared DHSs were counted after MACS2 peak calling [47]. The two replicates shared 14,401 DHSs (66% and 88% of the peaks in replicate 1 and 2, respectively; Table S2). We concluded that the results from the technical replicates were comparable. The reads from the two technical replicates were therefore pooled and treated as one biological replicate in the further analysis.

H3K4me3 ChIP-seq data were obtained from the NCBI database (SRX1073672; [61]). The quality filtering of the sequencing data was done in the same way as described in the BS-seq analysis section. The reads were aligned to the reference genome using BWA [107]. Non-uniquely mapped reads were filtered out with a MAPQ cutoff value of 20 using samtools [108]. Peaks were called for each biological replicate with a q-value cutoff of 0.001 using MACS2 [47]. During the peak calling, naked DNA digestion data and input control data were used as controls for DNase-seq and for ChIP-seq, respectively. Only peaks with 70% or larger overlap between replicates were kept for analysis. If there were three replicates, overlapping peaks in two replicates were identified first and then the third replicate was compared to the already-integrated peaks.

#### BS-seq

Raw data of genome-wide bisulphite sequencing experiments on wild-type B73 coleoptile shoot tissue (harvested five days after the start of germination) [35] was obtained from the NCBI database (GSE39232). FastX toolkit [109] was used to filter artefacts introduced by library construction such as linker and/or adaptor sequences, and to filter reads of which the qualities of more than 80% of the bases were lower than a threshold of Q20. The reads were trimmed based on their per-base sequence qualities and reads shorter than 70 bases after trimming were removed using PRINSEQ [110]. The read mapping to the reference genome and methylation base calling was performed using BS-seeker2 [111]. The LUMRs were identified for both CG and CHG data using MethylSeekR [31]. The threshold for percent methylation for the low methylated regions (LMRs) was set to 20%. MethylSeekR [31] defines unmethylated regions (UMRs) and LMRs; in this study, we combined both regions into one class, LUMRs. Any identified regions with more than or equal to 20% DNA methylation using bwtool [48] were further filtered out. For enhancer identification, regions with both low CG and low CHG methylation, which were identified using BEDtools [102], were called LUMRs. The methylation frequency at every mCG, mCHG and mCHH position was extracted for further analysis.

#### Characterisation of each dataset

Genomic regions were defined as follows: genic regions, exons and TEs were annotated according to the reference annotation. The annotated exons include the untranslated regions (UTRs). The entire genome, except for the genic regions, were called intergenic regions. Introns were genic regions excluding exons. Promoters were defined as the sequence 1 kb upstream and 200 bp downstream of TSSs. Flanking regions were defined as sequences 4 kb upstream from promoter regions and 5 kb downstream from the TTSs. Distal regions were intergenic regions that were not classified above. Uniquely mappable regions in the whole genome were identified using Uniqueome [42] for theoretical read lengths of 93 bp, which was the longest read possible for the ISAS uniqueome aligner (http://www.imagenix.com) to handle and closest to the actual read length (100 bp), allowing two mismatches. The ISAS uniqueome aligner performs all-against-all sequence alignment with a given read length (93 bp in this case) and deduces percent uniqueness for each nucleotide position based on the percentage of reads mapped to this position that are uniquely mapping at this location. In this study, uniquely mappable regions showed 90% or higher uniqueness. The number of uniquely mappable base pairs within each genomic region was counted using BEDtools [102] and plotted using the plotrix package [112] in R [113].

The total lengths of each genomic region in Mbs and the numbers of features (DHS, H3K9ac and LUMR) overlapping with the defined genomic regions were counted using BEDtools [102] and plotted using R [113].

For correlations between gene expression levels and DNase hypersensitivity or H3K9ac enrichment, first the genes were binned based on their expression levels in RPKM from the lowest (bin 0) to the highest (bin 6). Bin 0 contains all the genes with no and lower than 1 RPKM expression. The other six bins were defined so that each bin contained exactly the same number of genes. The average intensities of DNase hypersensitivity and H3K9ac enrichment in RPM over genic regions were calculated using bwtool [48] and plotted using R [113].

### Data integration

#### Candidate identification

The enhancer prediction in this study was focused on active enhancers. The DNase I hypersensitivity, H3K9ac enrichment and LUMR data were integrated. All LUMRs that overlap with DHSs and H3K9ac (Fig. 2), excluding the ones overlapping with genes and promoter regions, and the numbers of candidates overlapping with TEs and CNSs were selected and counted using BEDtools [102]. The CNS coordinate data were extracted from published rice v6 versus maize v2 data [66] and the coordinates were converted from v2 to v4 using Assembly Converter available on Ensembl Plants [40].

#### TE enrichment analysis

TE annotations are available at the Gramene database (ftp://ftp.gramene.org/pub/gramene/CURRENT_RELEASE/data/gff3/zea_mays/repeat_annotation/) [67] and TE families have been named according to the guidelines described at the Maize TE database (http://maizetedb.org/cgi-bin/cgiwrap/maize/TE_show_family.cgi?do_table = 1).

To prepare the annotation file, nested TE insertions were resolved using RTrackLayer [114] in R [113]. Bedtools intersect [102] was then used to find overlaps between enhancer candidate coordinates and TE coordinates. Enhancers candidates that were at least 80% contained within a single TE were selected for further analysis. To create a baseline for the number of TEs that could contain an intergenic enhancer candidate, the full list of TEs was filtered for elements not contained within introns and that are longer than 635 bp, long enough that the enhancer candidates at the 20th percentile by length could overlap 80% of a TE. The filtered TE set was used as the baseline for number of elements within families containing enhancer candidates (Additional file 4: Dataset 3) and for comparisons between TEs with and without enhancer candidates. Conserved sequence motifs were identified using four de novo motif discovery tools, HOMER, MEME, DREME and RSAT plants [68–71] and enhancer candidates, the TE family RLG00010 and randomly selected intergenic sequences of the corresponding size were scanned for the identified motifs using FIMO [77].

#### Heatmap plot

For DNase I hypersensitivity and H3K9ac enrichment, RPM signal tracks were generated from pooled data during peak calling using MACS2 [47]. DNase I hypersensitivity, H3K9ac enrichment and transcript coverage data, methylation frequency data and TE annotation data (0 = absence, 1 = presence of TEs) were converted to BigWig files using wigToBigWig tool [115]. For the DNA methylation data, methylation frequency over 100-bp fixed-windows were calculated using bwtools [48].

The data on DNase I hypersensitivity, H3K9ac enrichment, mCG, mCHG and mCHH levels, TE presence and transcript levels were extracted for each DHS and its 1-kb flanking regions in our candidate list using bwtool [48]. The DHSs were clustered based on H3K9ac enrichment with k-means clustering, re-ordered, and all the datasets were plotted according to the order defined based on H3K9ac k-means clustering using the gplots package [116] in R [113]. For the heatmap profile at TSSs, 429 DHSs mapped at TSSs in V2-IST and 1400 in husk were randomly selected and heatmaps were generated in the same manner as for the DHSs in the candidates. To make the heatmaps comparable, for DHSs at TSSs, the same number of DHSs were selected as the number of DHSs in candidates in the two tissues.

#### Genomic feature profiling at DHSs in enhancer candidates and TSSs

To understand the behaviour of H3K9ac enrichment and DNA methylation around DHSs at our potential candidate regions, average profiles were generated. First, all the intergenic DHSs were taken. For each DHS, H3K9ac enrichment values 300 bp upstream and downstream were extracted using bwtool [48] and the end with higher H3K9ac enrichment was defined as 3' end of the DHS. Using bwtool [48], the average RPM for DNase I hypersensitivity and H3K9ac enrichment and methylation frequencies at CG, CHG and CHH were calculated at the intergenic DHSs and their flanking regions. The values were plotted using R [113]. Average profiles for TSSs were generated in the similar manner except the DHSs were oriented based on their gene strand. For generating average plots for each category, the DHSs were first binned by the categories and average values were calculated for each bin.

#### Candidate ranking

Once enhancer candidates were identified, they were ranked according to their presumed tissue-specificity. We assumed that the tissue-specificity of an enhancer is correlated to its DNase I hypersensitivity and H3K9ac enrichment. Therefore, the tissue-specificity of each candidate was determined using the largest differences in DNase I digestion sensitivity and H3K9ac enrichment between the two tissues (Fig. 2). For each candidate, for both the DNase hypersensitivity and H3K9ac enrichment separately, the intensity differences in the candidate region were calculated from the signal tracks and the largest values were taken as the difference using bwtool [48]. The candidates were then ranked based on the differences in DNase I hypersensitivity and H3K9ac enrichment independently and the DNase I and H3K9ac rankings were summed for each candidate. Then, the enhancer candidates were re-ranked based on the sum. The V and H numbers provided in the Additional files 2 and 3 show the final ranking after the summation. For *p* value calculation, two (or three for H3K9ac husk data as it had three replicates) lists of numbers (1 to 398 for V2-IST, 1 to 1320 for husk tissue, the same number as the numbers of candidates) were generated. Random combinations of two (or three) numbers were summed and re-ranked according to the sum 1000 times to create lists of theoretical summation scores for each ranking. The frequency occurrence of the value less than or equal to the real data in the theoretical score list was computed and provided as *p* values.

#### Linking enhancer candidates to potential target genes

Enhancer candidates were linked to putative target genes based on the defined tissue-specificity of candidates and expression data of nearby genes. The assumption was that an enhancer targets its closest upstream or downstream gene. First, gene expression levels and the statistical significance of their differential expression data from Cuffdiff [86] were linked to the gene coordinate data. The closest upstream and downstream genes were identified for each candidate using BEDtools [102]. For tissue-specific candidates, significantly differentially expressed genes were identified first, then the tissues in which the genes were expressed higher were identified. When the tissue-specific gene expression levels matched with the tissue-specificity of the candidate, the gene(s) was linked to the candidate. For example, if one of the candidates was determined as V2-IST-specific and the upstream gene had higher expression in V2-IST than in husk, we concluded that the candidate most probably regulates its upstream gene. For shared candidates, adjacent genes being expressed in both tissues were associated.

## Additional files

Additional file 1: Figure S1. The tissues used in this study. **Figure S2.** Reproducibility of RNA-seq data. **Figure S3.** Randomised distributions of features over genomic regions within the uniquely mappable part of the genome. **Figure S4.** (A) The size distributions of the different features in base pairs and (B) the distributions of the gene expression levels in the two tissues. **Figure S5.** Characteristics of enhancer-overlapping TEs. **Figure S6.** Examples of (A, B) enhancers that contain TEs and (C, D) TEs that contain an enhancer. **Figure S7.** Example of data on *tb1* enhancer. **Figure S8.** Asymmetric H3K9ac enrichment at candidate DHSs. **Figure S9.** Average profiles of the enhancer candidates in V2-IST and husk for each category. **Figure S10.** Heatmaps of chromatin, DNA and transcript features at TSSs. **Figure S11.** Average profiles of randomly selected TSSs in V2-IST and husk for each category. **Figure S12.** Comparison of expression levels between genes and enhancer candidates in V2-IST and husk. **Figure S13.** Heatmaps and average profiles of H3K9ac and H3K4me3 at (A, B, D) candidates and (A, C, E) TSSs. **Figure S14.** ChIP-qPCR data on H3K9ac and H3K4me1 enrichment at enhancer candidate regions. **Figure S15.** DNase I experiments resulting in the libraries sequenced and analysed in this study. **Table S1.** Sequencing datasets generated in this study. **Table S2.** Number of DHSs that overlap between replicates. **Table S3.** Number of H3K9ac peaks that overlap between replicates. **Table S4.** Number of TEs (for RLG family with and without candidates) and enhancers that carried the GGCCCA motif. Supplemental Methods. (PDF 2734 kb)

Additional file 2: List of enhancer candidates in V2-IST. The columns indicate from left to right: chromosome number, start coordinate, end coordinate, candidate ID, DNase intensity ranking, DNase ranking *p* value, H3K9ac intensity ranking and H3K9ac ranking *p* value, absence or presence of CNSs. (XLSX 30 kb)

Additional file 3: List of enhancer candidates in husk. The columns indicate from left to right: chromosome number, start coordinate, end coordinate, candidate ID, DNase intensity ranking, DNase ranking *p* value, H3K9ac intensity ranking and H3K9ac ranking *p* value, absence or presence of CNSs. (XLSX 86 kb)

Additional file 4: Number of enhancer candidates overlapping with TEs in individual TE families. TE Superfamily column states which TE superfamily the TEs belong to, the TE family column provides the TE family IDs, the family members column lists the total number of elements remaining after removing short and intronic TEs (see methods). For V2-IST, Husk and total enhancer candidates, the number of candidates overlapping at least 80% with TEs within a family and the percent of TEs overlapping with enhancer candidates within the particular family are shown. In the p-val column, the *p* values calculated using the binomial test with Bonferoni correction are given for the total number of enhancer-containing TEs in a given TE family. The cells highlighted in yellow indicate the TE families significantly enriched for enhancer-containing TEs. (XLSX 26 kb)

Additional file 5: List of tissue-specific and shared candidates and their linked genes. Columns from the left to right indicate: candidate chromosome location and coordinates and ID followed by chromosome coordinates, IDs, orientation, expression levels in V2-IST and husk of upstream and downstream adjacent genes. The significance of differential expression for flanking genes is indicated. NA means that the upstream or downstream gene did not fulfil the requirement to be associated as a target gene. (XLSX 171 kb)

Additional file 6: List of primers used in ChIP-qPCR experiments. The first and second column indicate the loci the primers anneal to, and the name of the primer pairs, respectively. The right column indicates the sequences of the forward (F:) and reverse (R:) primers for each pair. (XLSX 12 kb)

## Abbreviations

BS: Bisulphite conversion
ChIP: Chromatin immunoprecipitation
CNS: Conserved non-coding sequence
DHS: DNase I hypersensitive site
eRNA: enhancer RNA
LTR: Long terminal repeat
LUMR: Low and unmethylated DNA region
ncRNA: Non-coding RNA
seq: High-throughput sequencing
TE: Transposable element
TF: Transcription factor
TIR: Terminal inverted repeat.
TSS: Transcriptional start site
TTS: Transcription termination site
V2-IST: Inner-stem tissue of V2 stage seedlings
V5-IST: Inner stem tissue of V5 stage seedlings
