# Supplementary material for: Genome-wide mapping of transcriptional enhancer candidates using DNA and chromatin features in maize
Source: Genome Biol. 2017 Jul 21;18:137. doi: 10.1186/s13059-017-1273-4 (PMC5522596; doi:10.1186/s13059-017-1273-4)
Supplement: Supplementary file 1 — The tissues used in this study. Figure S2. Reproducibility of RNA-seq data. Figure S3. Randomised distributions of features over genomic regions within the uniquely mappable part of the genome. Figure S4. (A) The size distributions of the different features in base pairs and (B) the distributions of the gene expression levels in the two tissues. Figure S5. Characteristics of enhancer-overlapping TEs. Figure S6. Examples of (A, B) enhancers that contain TEs and (C, D) TEs that contain an enhancer. Figure S7. Example of data on tb1 enhancer. Figure S8. Asymmetric H3K9ac enrichment at candidate DHSs. Figure S9. Average profiles of the enhancer candidates in V2-IST and husk for each category. Figure S10. Heatmaps of chromatin, DNA and transcript features at TSSs. Figure S11. Average profiles of randomly selected TSSs in V2-IST and husk for each category. Figure S12. Comparison of expression levels between genes and enhancer candidates in V2-IST and husk. Figure S13. Heatmaps and average profiles of H3K9ac and H3K4me3 at (A, B, D) candidates and (A, C, E) TSSs. Figure S14. ChIP-qPCR data on H3K9ac and H3K4me1 enrichment at enhancer candidate regions. Figure S15. DNase I experiments resulting in the libraries sequenced and analysed in this study. Table S1. Sequencing datasets generated in this study. Table S2. Number of DHSs that overlap between replicates. Table S3. Number of H3K9ac peaks that overlap between replicates. Table S4. Number of TEs (for RLG family with and without candidates) and enhancers that carried the GGCCCA motif. Supplemental Methods. (PDF 2734 kb) [file 13059_2017_1273_MOESM1_ESM.pdf]

Supplemental Figures, Tables and Methods

(A) V2-IST

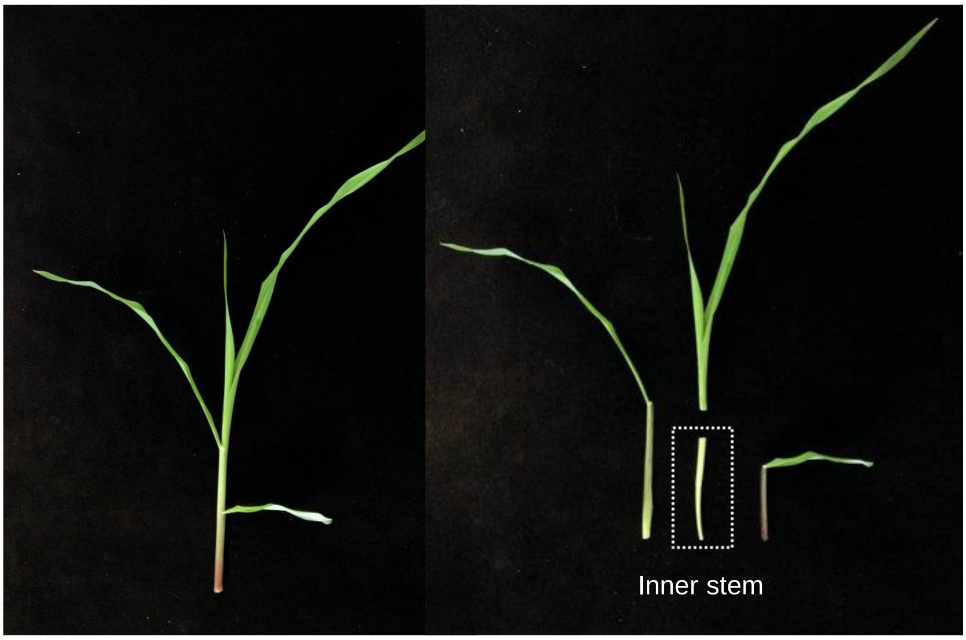

(B) Husk

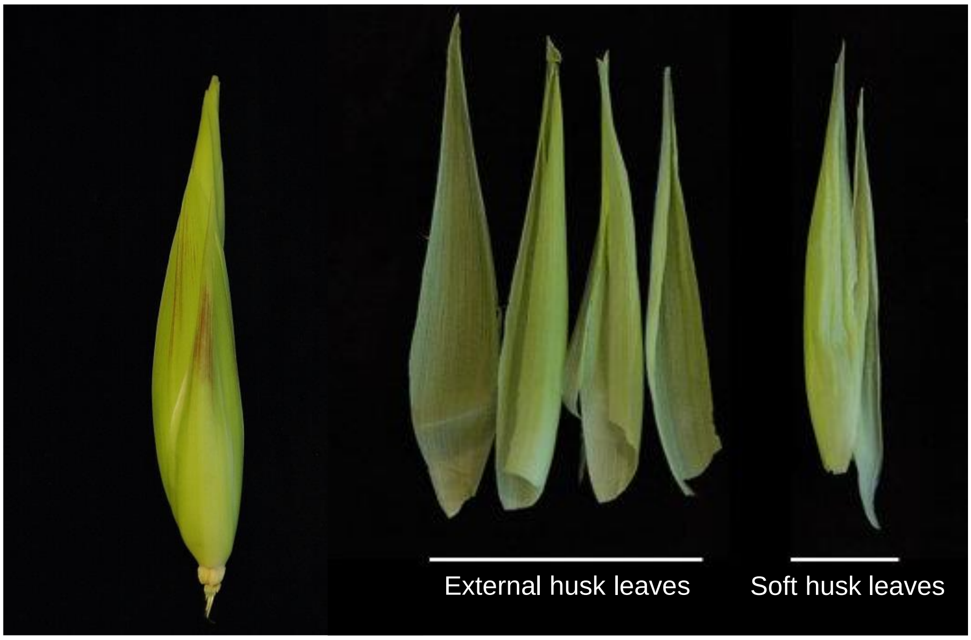

Figure S1. The tissues used in this study. (A) Inner stem tissue (at the right) isolated from V2 seedling (left) and (B) soft husk leaves (at the right) isolated from maize husk (left).

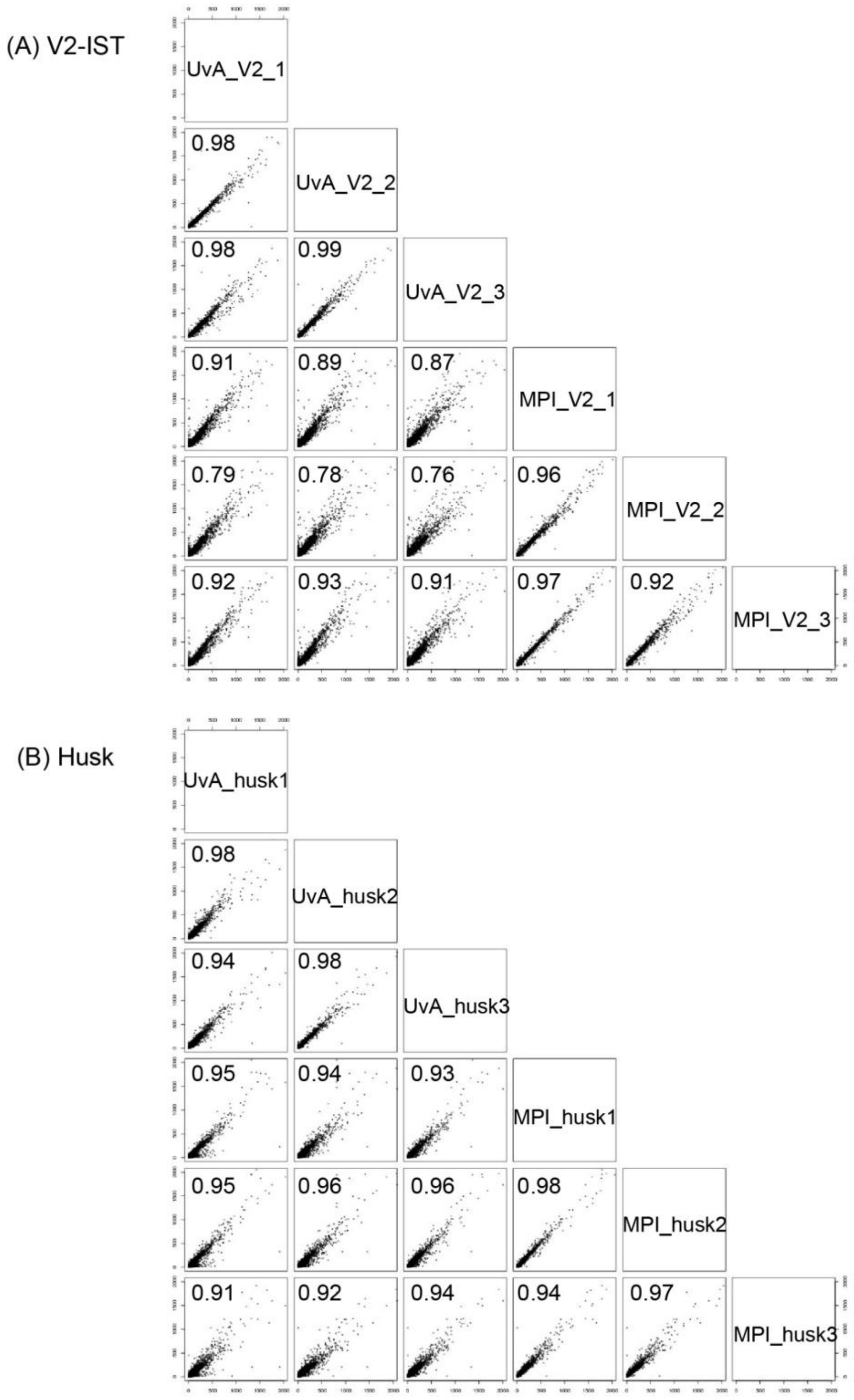

Figure S2. Reproducibility of RNA-seq data. Gene expression levels (in read counts) were plotted for all genes. Read counts were normalised by the total number of reads in the respective replicates. (A) V2-IST samples, (B) Husk samples. Pearson correlation coefficients are given in each scatter plot.

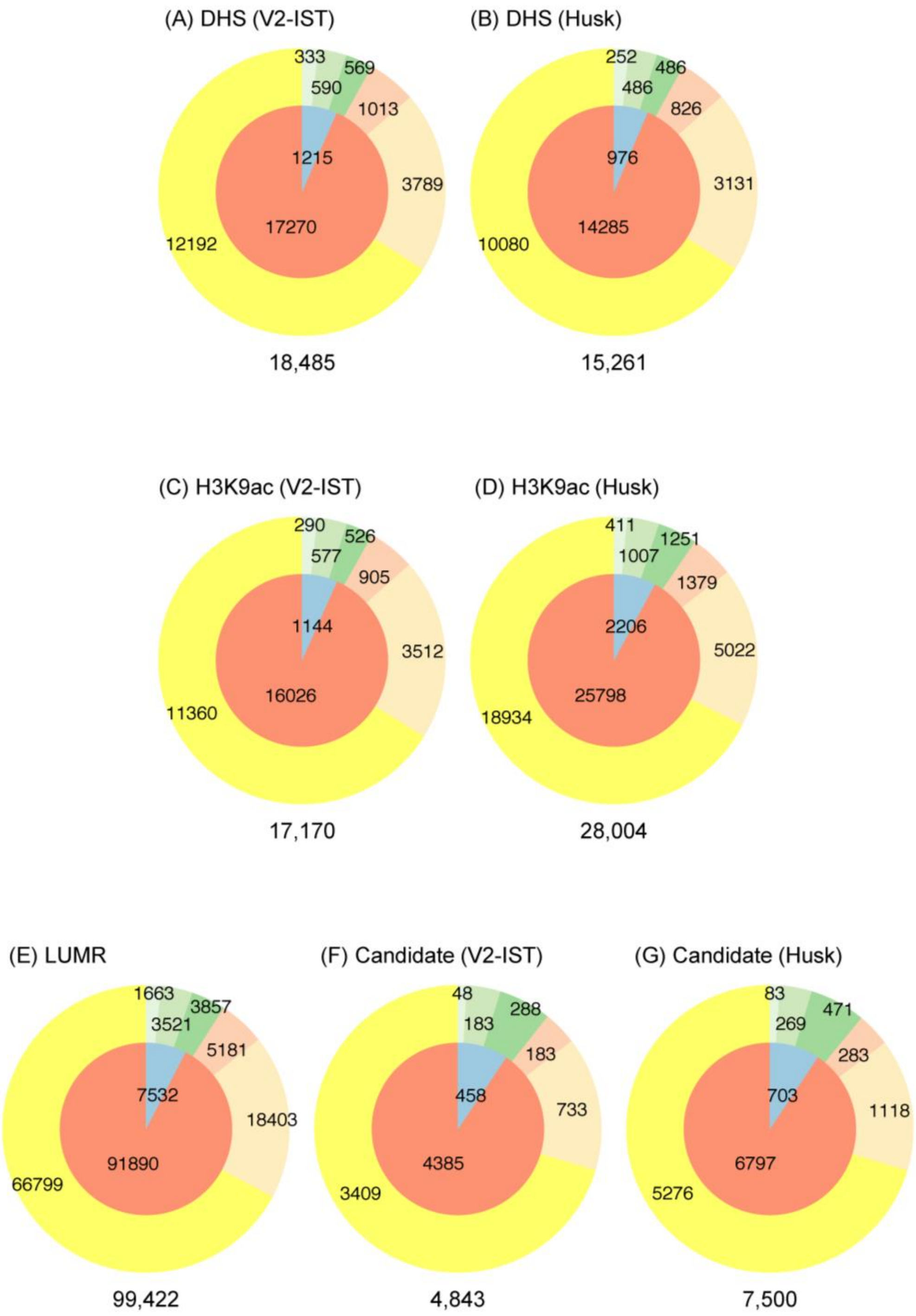

Figure S3. Randomised distributions of features over genomic regions within the uniquely mappable part of the genome. Distributions of (A, B) DHSs, (C, D) H3K9ac (E) LUMRs and (F, G) enhancer candidates over the different genomic regions. The numbers below the pie charts are the total numbers of the indicated features in the genome. Inner ring: intergenic (red) and genic (blue); outer ring: intergenic TEs (yellow), distal (sand), and flanking (peach) regions, promoters (dark green), exons (light green), and introns including intronic TEs (mint) as depicted in Figure 3A.

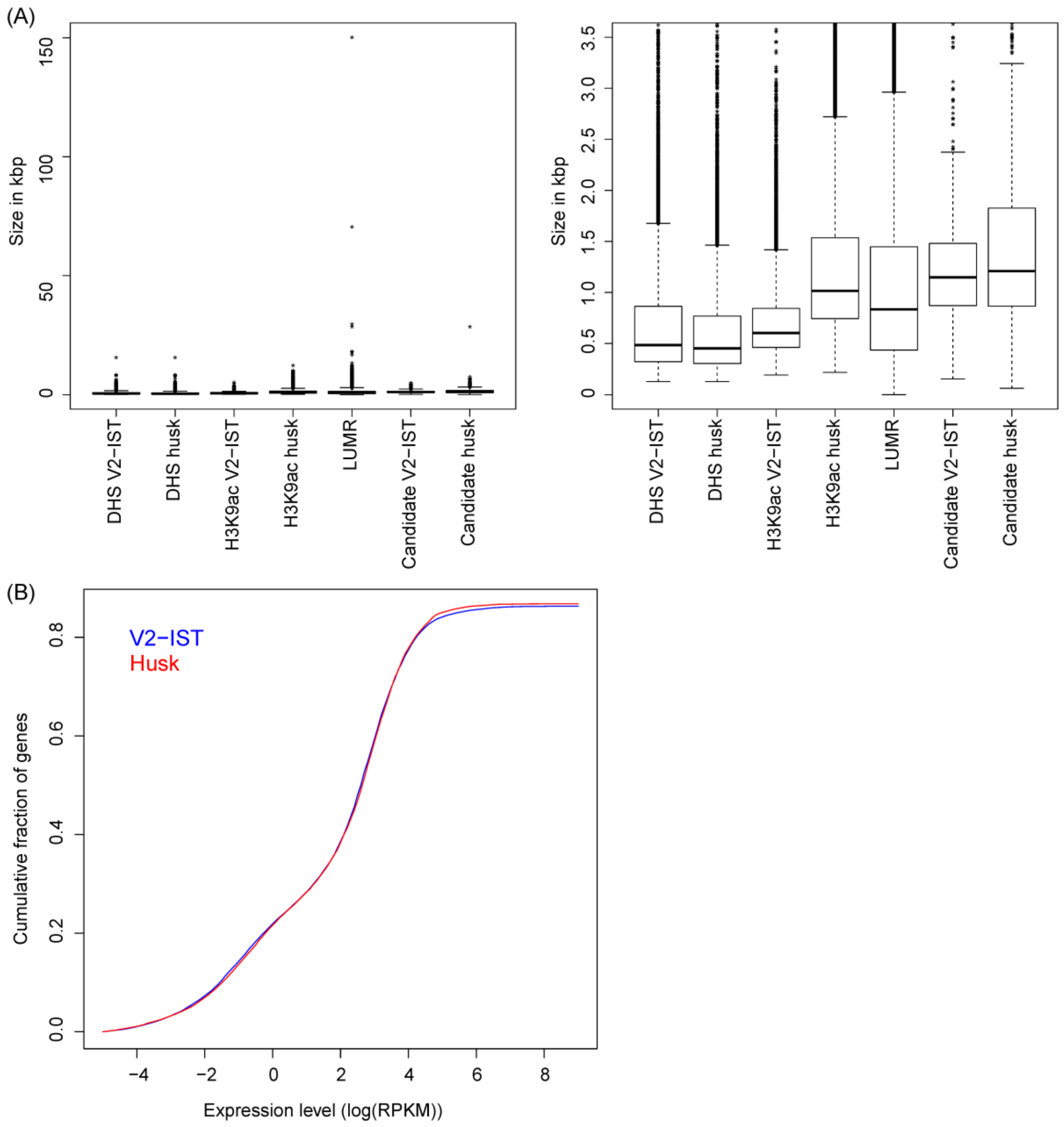

Figure S4. Characteristics of genomic features and distribution of gene expression levels. (A) Size distributions (in kilobase pairs) of chromatin and DNA features as indicated on x-axis categories. Left panel shows full ranges including all outliers, right panel a zoom into the data. Boxes include second and third quartile, the median is indicated by a horizontal black line. Whiskers extend the interquartile range by 1.5, values beyond are plotted as outliers. (B) Cumulative fraction of genes contributing to total expression quantified as sum of normalized (RPKM) RNA-seq reads in V2-IST (blue) and husk (red).

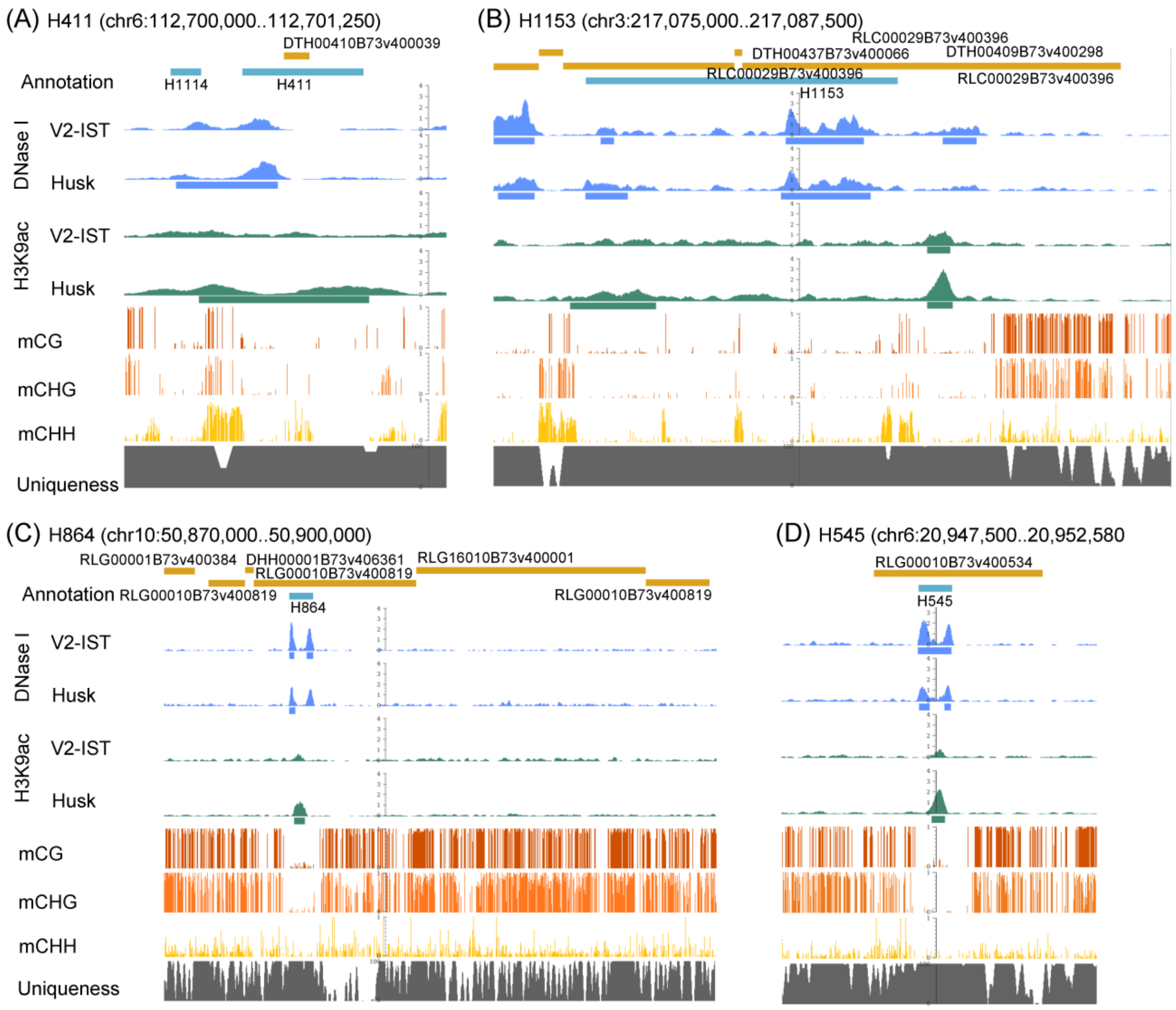

Figure S5. Examples of (A and B) enhancers that contain TEs and (C and D) TEs that contain an enhancer. Jbrowse tracks show from the top: AGPv4 TE (ochre) [1] and candidate enhancer (turquiose) annotation (H stands for husk candidate), DNase-seq (blue) and H3K9ac (green) coverage, and peak position (indicated as bars) in V2-IST and in husk, mCG, mCHG and mCHH levels [2] and Uniqueness as mappability in percentage. (A, B) H411 and H1153 containing DTH00410 and DTH00409, respectively, both belong to the TIR TE order. (C, D) RLG0010 family members containing enhancer candidates H864 and H545. Note that tracks for accessibility and H3K9ac coverage represent pooled replicates. Peaks at those tracks might not be present in each replicate, affecting enhancer candidate prediction.

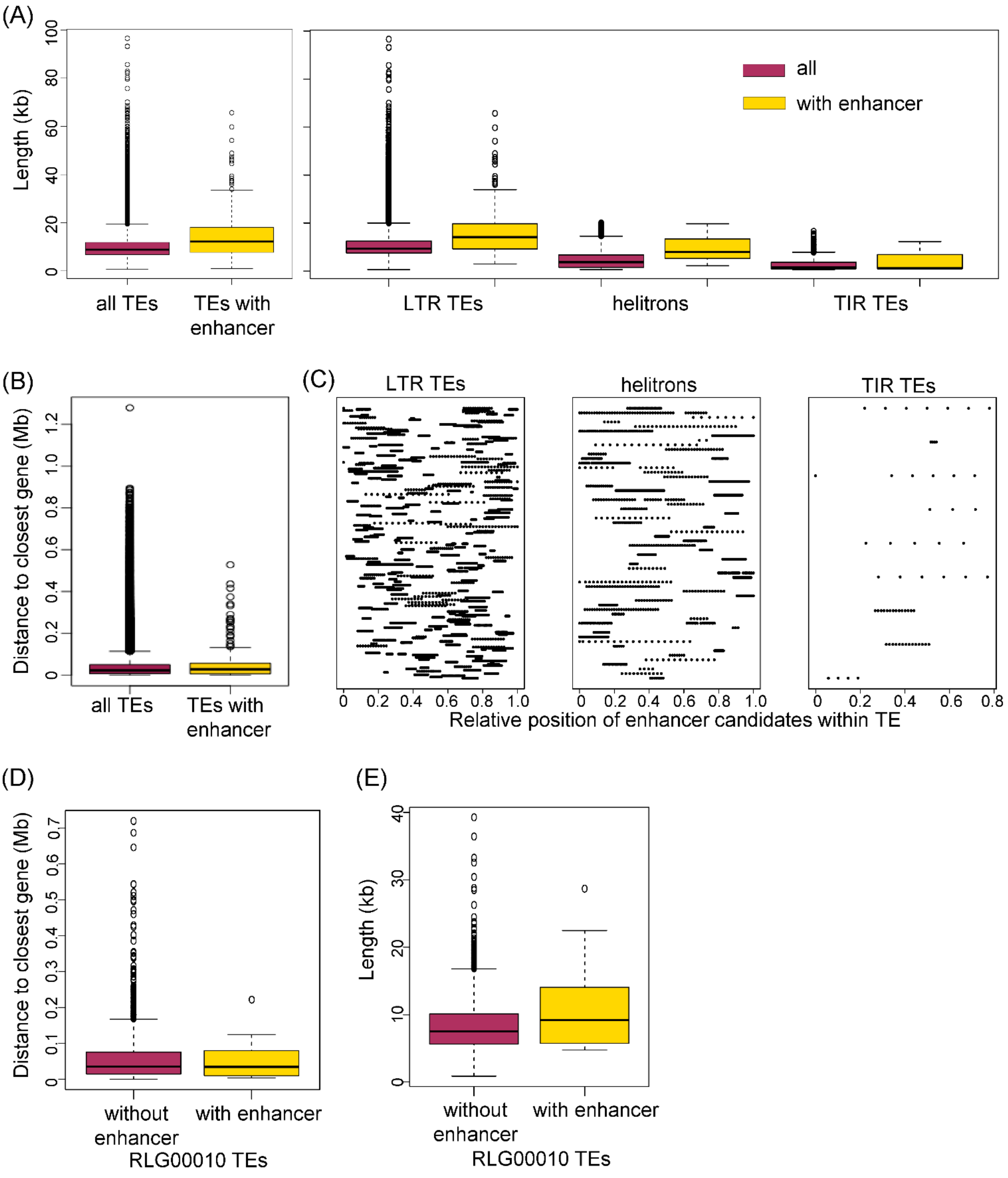

Figure S6. Characteristics of enhancer-overlapping TEs. Comparison of (A) the lengths of all TEs and per major TE order. (B) Distribution of distance between TEs and their closest flanking genes. (C) Relative locations of enhancer candidates within TEs per major order. (D, E) Comparison of RLG00010 family members with and without enhancer candidate for (D) distances to their closest flanking genes and (E) TE lengths. Boxes include second and third quartile, the median is indicated by a black line. Whiskers extend the interquartile range by 1.5, values beyond are plotted as outliers.

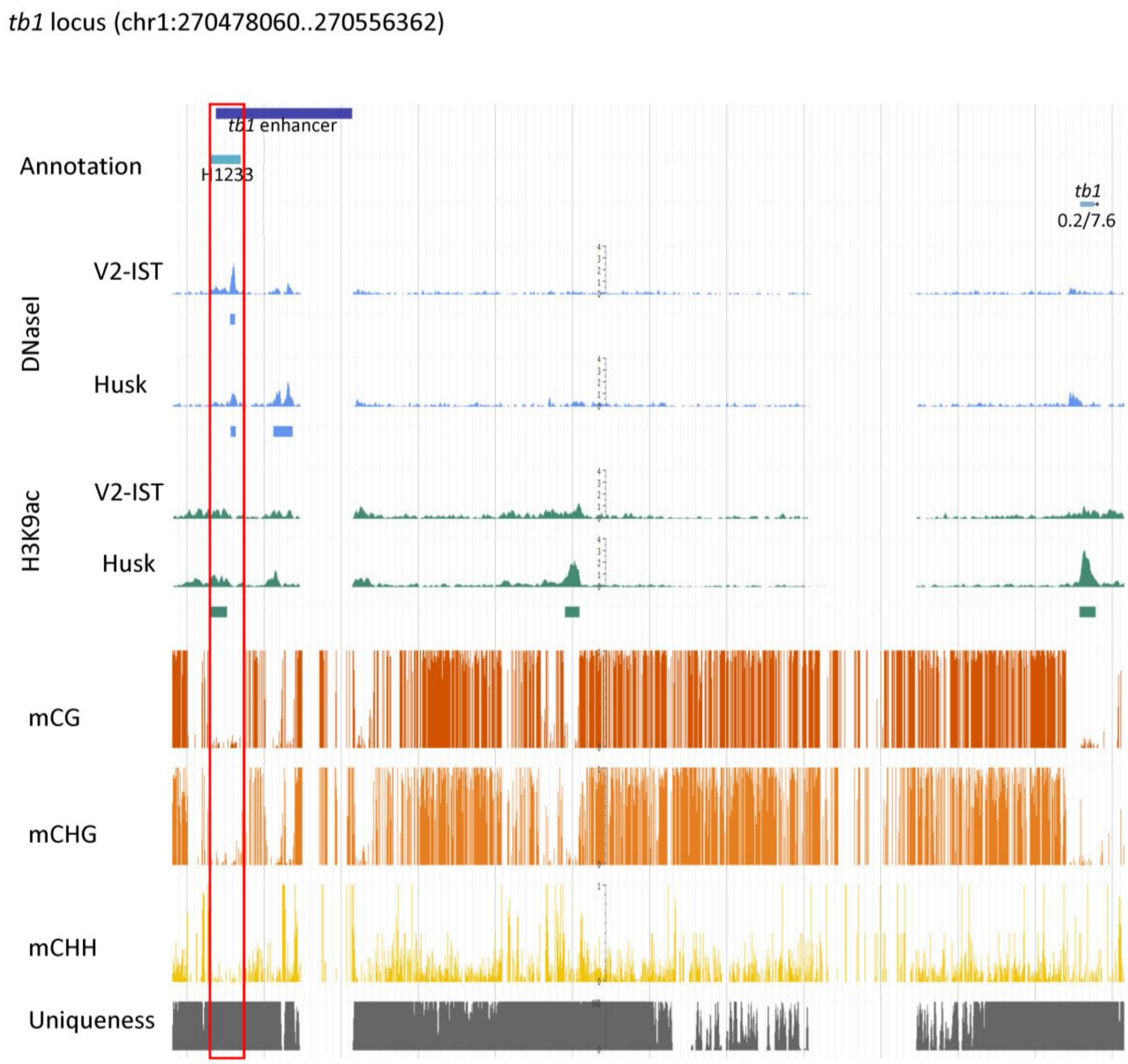

Figure S7. Example of data on *tb1* enhancer. From the top: AGPv4 genome annotation [1], candidate annotation from our prediction (H stands for husk candidate), DNase-seq and H3K9ac ChIP-seq read coverage and data on peak position (indicated as bars) in V2-IST and in husk, mCG, mCHG and mCHH levels [2] and unique mappability in percentage. The number under the gene name indicates gene expression levels (V2-IST/husk). The dark blue bar in the gene annotation track indicates the previously annotated *tb1* enhancer. The vertical red box indicates the enhancer candidate identified in this study. Note that tracks for accessibility and H3K9ac coverage represent pooled replicates. Peaks at those tracks might not be present in each replicate, affecting enhancer candidate prediction.

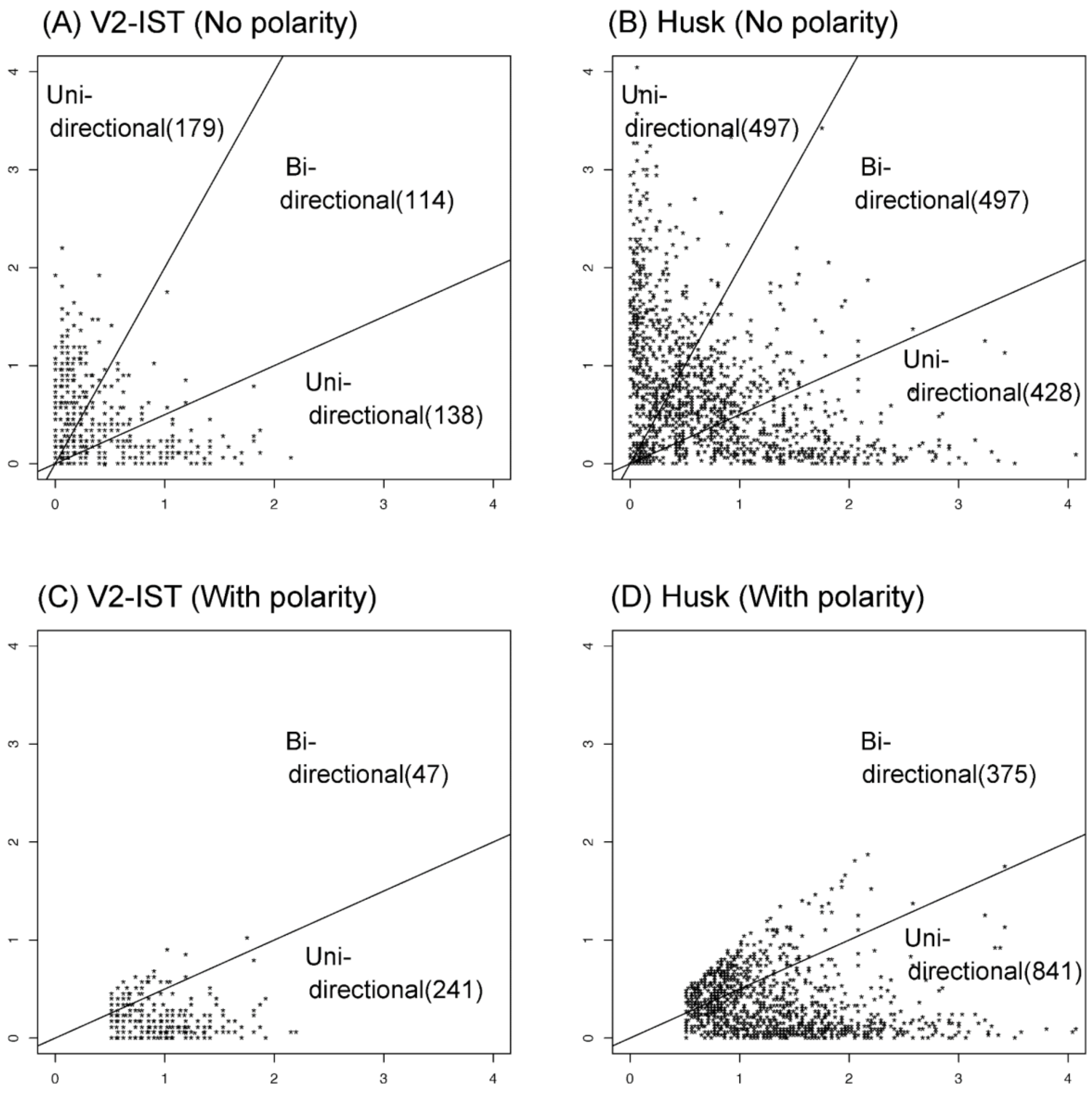

Figure S8. Asymmetric H3K9ac enrichment at candidate DHSs. Distributions of H3K9ac enrichment 300 bp up- and downstream from the boundaries of DHSs in 431 V2-IST and 1,437 husk enhancer candidates (A, B) before and (C, D) after orientating the candidates based on the H3K9ac enrichment values 300 bp up- and downstream from the DHS boundaries; the higher H3K9ac values were plotted on the x-axis, the lower on the y-axis. In C and D, the number of data points below the line were 241 in V2-IST and 841 in husk, indicating asymmetrical H3K9ac enrichment.

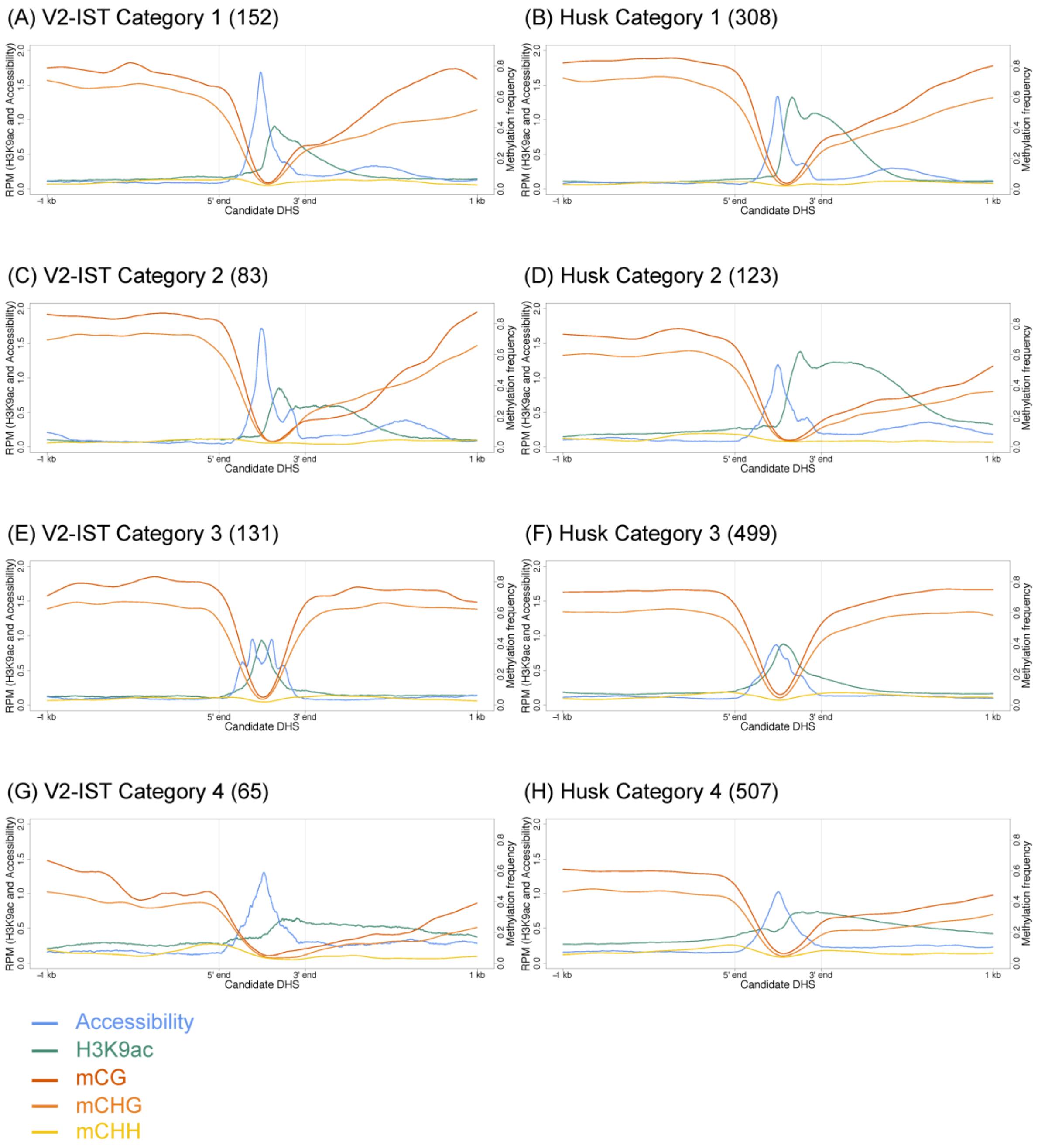

Figure S9. Average profiles of the enhancer candidates in V2-IST and husk for each category. Average signal intensities of DNase I hypersensitivity, H3K9ac enrichment in RPM and DNA methylation levels in methylation frequency [2] at DHSs and their 1 kb flanking regions. The categories, identified in heatmaps by clustering DHSs on H3K9ac enrichment data using a k-means (k=4) algorithm (Figure 5), were numbered from 1 to 4 from the top of the figure to the bottom. The DHSs were oriented based on the flanking signals of H3K9ac enrichment (higher H3K9ac enrichment on the 3' side). The various sizes of DHSs have been scaled to an equal size. The numbers between brackets represent the number of enhancer candidates in the category and tissue indicated.

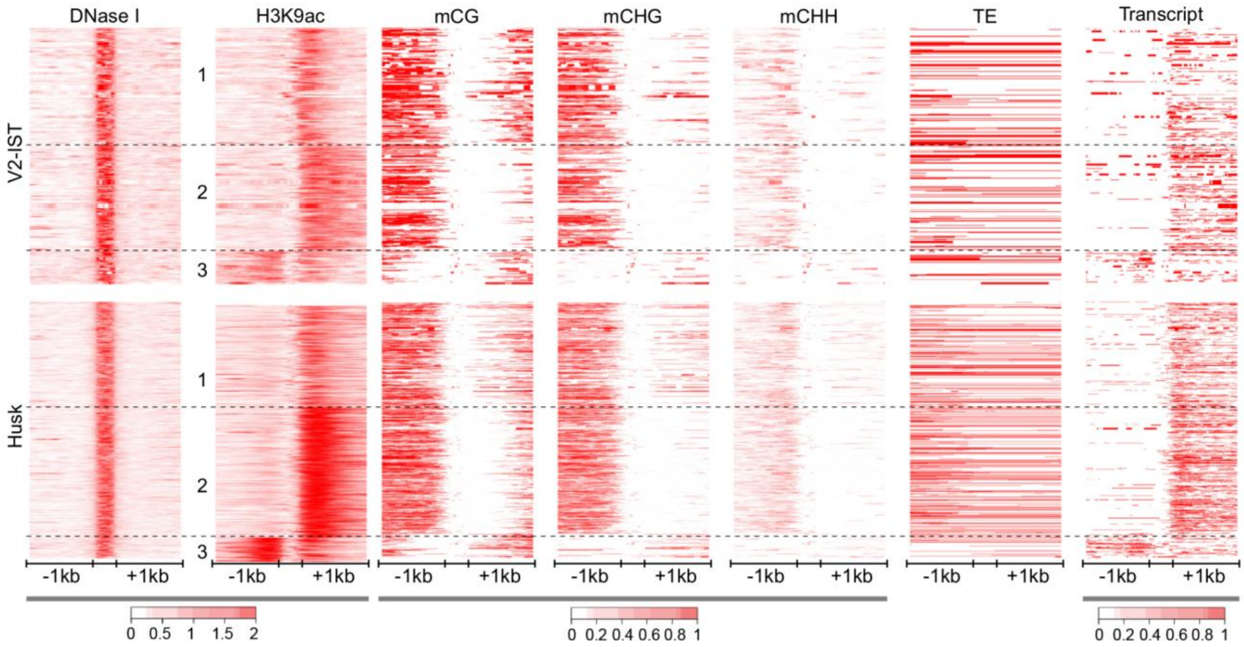

Figure S10. Heatmaps of chromatin, DNA and transcript features at TSSs. DNase I hypersensitivity, H3K9ac enrichment, mCG, mCHG and mCHH levels [2], presence of TEs [1], and transcript levels at and around (+/- 1 kb) DHSs at TSSs (431 and 1,437 in V2-IST and husk, respectively). The selected regions were oriented according to the 5' to 3' orientation of the genes. The various sizes of DHSs have been scaled to an equal size. The colour scales are in RPM for DNase I hypersensitivity, H3K9ac enrichment and transcript levels, and in methylation frequency (0-1) for DNA methylation. For TEs, red and white shows the presence or absence of TEs, respectively. The DHSs were clustered on the H3K9ac enrichment data using a k-means (k=3) clustering algorithm.

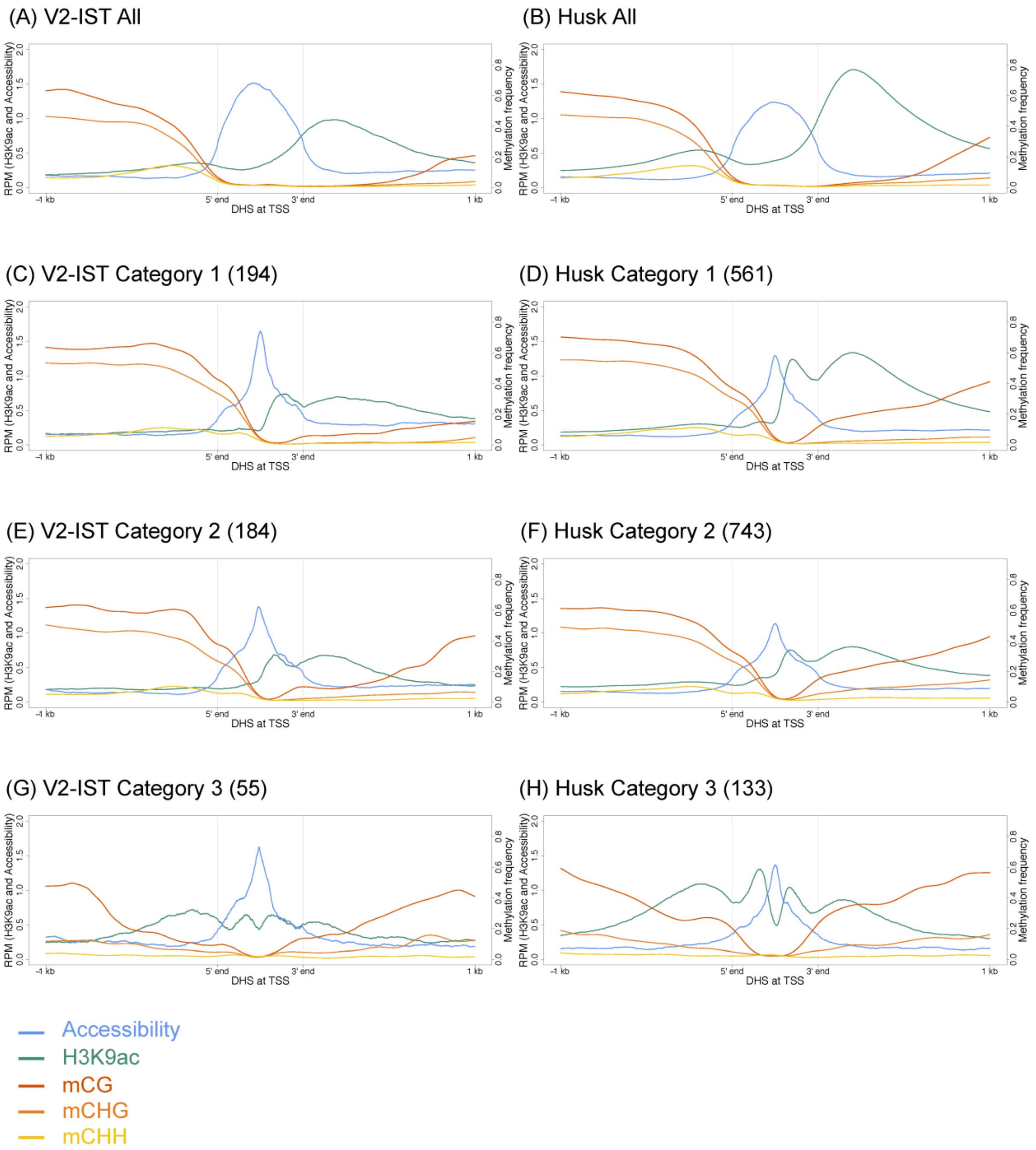

Figure S11. Average profiles of randomly selected TSSs in V2-IST and husk for each category. 431 V2-IST and 1,437 husk TSSs overlapping with DHSs and H3K9ac were randomly selected. The DHSs at the TSSs were oriented according to the 5' to 3' orientation of the genes. (A, B) Average signal intensities of DNase I hypersensitivity, H3K9ac enrichment in RPM and DNA methylation levels in methylation frequency [2] at DHSs and their 1 kb flanking regions. (C-H) The three categories, identified in heatmaps by clustering DHSs on H3K9ac enrichment data using a k-means (k=3) algorithm (Figure S10), were indicated.

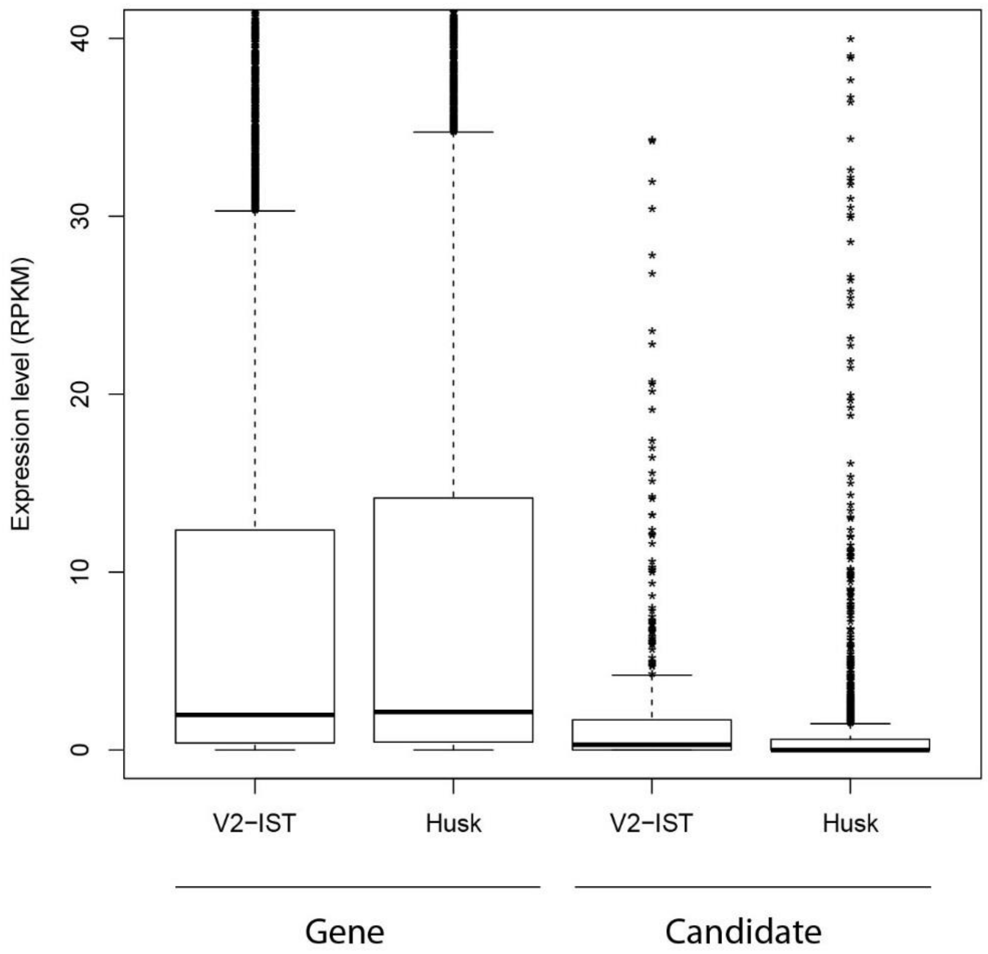

Figure S12. Comparison of expression levels between genes and enhancer candidates in V2-IST and husk. Boxplot showing expression levels (RPKM) for genes and enhancer candidates in V2-IST and husk tissue. Boxes include second and third quartile, the median is indicated by a black line. Whiskers extend the interquartile range by 1.5, values beyond are plotted as outliers.

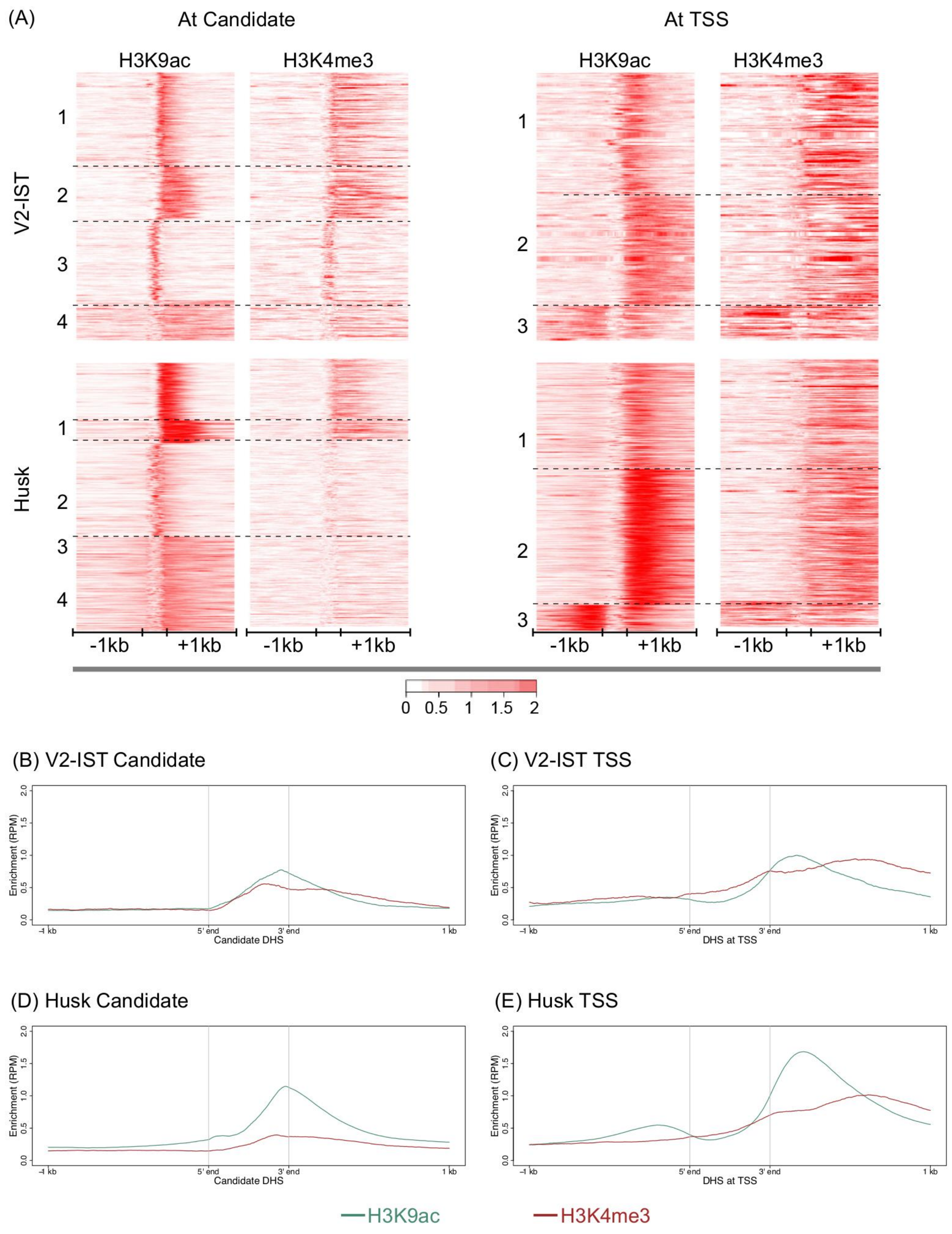

Figure S13. Heatmaps and average profiles of H3K9ac and H3K4me3 at (A, B, D) candidates and (A, C, E) TSSs. (A) The H3K9ac clustered data in V2-IST and husk tissue are the same data as shown in Figure 5 and S10. In addition, H3K4me3 enrichment data [3] at the same loci was plotted in the same manner as for H3K9ac. (B-E) Average H3K9ac and H3K4me3 enrichment signal intensities are shown.

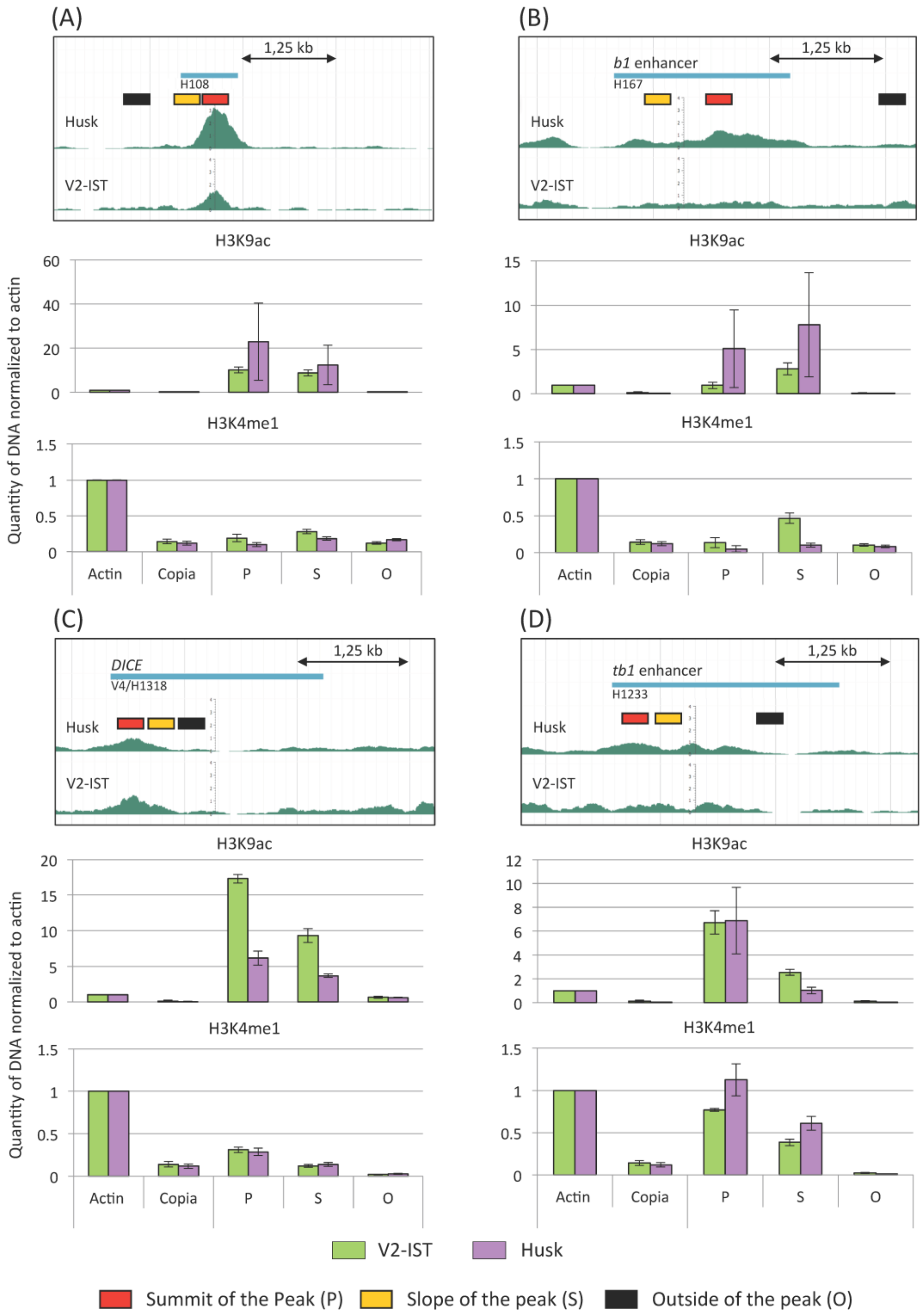

Figure S14. **ChIP-qPCR data of H3K9ac and H3K4me1 enrichment at enhancer candidate regions.** Genome browser view and levels of H3K9ac and H3K4me1 enrichment at (A) enhancer candidate H108, (B) *b1* enhancer (H167), (C) DICE (V4/H1318), and (D) *tb1* enhancer (H1233). The genome browser view shows H3K9ac enrichment as detected by ChIP-seq at each candidate for both V2-IST and husk. The sequences examined by qPCR are indicated by coloured boxes. Red and yellow and black boxes indicate the summits (P) and slopes of H3K9ac peak regions (S), respectively; black boxes indicate regions without H3K9ac enrichment (O). Below the top panels, the levels of enrichment for H3K9ac and H3K4me1 in V2-IST (green bars) and husk (purple bars) at the regions of interest relative to those at the *actin* locus are shown. Error bars indicate the standard error for three biological replicates.

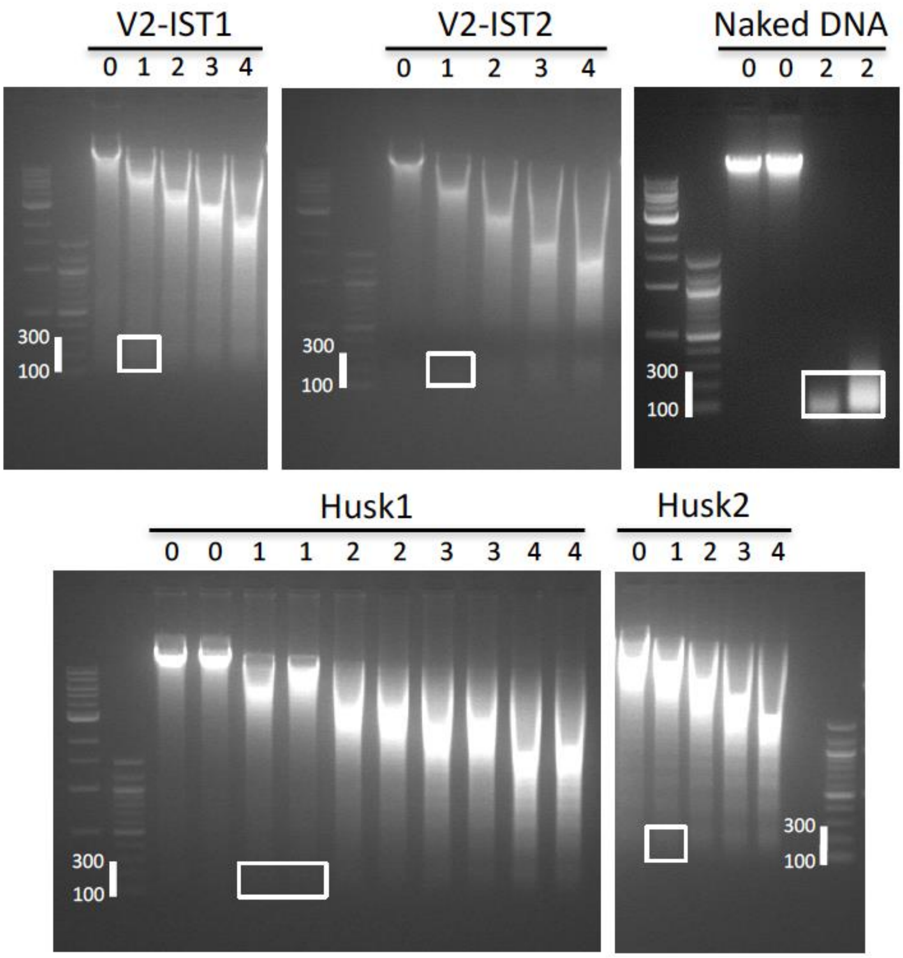

Figure S15: DNase I experiments resulting in the libraries sequenced and analysed in this study. Agarose gels stained with ethidium bromide displaying the DNA derived from DNase I digestions performed to generate the libraries sequenced: V2-IST1, V2-IST2, Husk1, Husk2, and Naked DNA. The concentrations of DNase I used were 0, 50, 100, 150, 200 U/ml, labelled 0, 1, 2, 3, and 4, respectively. The parts of the gels used to extract DNA for library preparation are indicated by white rectangles. The white vertical bars on the ladders indicate the size of the gel fractions selected (100 to 300 bp).

Table S1. Sequencing data sets generated in this study.

Library type	Tissue	Location	Replicate	Sequenced reads	After QC	Mapped reads	Unique reads
DNase-seq	V2-IST	MPIPZ	R1	30812785	30116531	29532397	12122567
		MPIPZ	R2	33388567	32822974	32313592	15608747
	Husk	MPIPZ	R1	55822602	53776042	52825014	25502270
		MPIPZ	R2	23749057	23147597	22719770	11116238
	Naked DNA control	MPIPZ	R1	49818293	46685455	46632980	19755778
H3K9ac ChIP-seq	V2-IST	UvA	R1	33814716	32691927	32650532	17963821
		UvA	R2	31670342	30769382	30734592	16765151
	Husk	UvA	R1	20941827	19521943	19371074	11584782
		UvA	R2	35359303	33755877	33682685	19579115
		UvA	R3	36216377	35035878	34982138	20765127
	V2-IST control	UvA	R1	51720348	49088635	49034975	23212740
		UvA	R2	58057106	54148015	54085846	25517430
	Husk control	UvA	R1	40617517	37966727	37914051	17948484
		UvA	R2	37565941	34715628	34665718	16379554
RNA-seq	V2-IST	MPIPZ	R1	16626209	16525399	14305067	13383964
		MPIPZ	R2	15830192	15722928	12959746	12094542
		MPIPZ	R3	14684139	14596625	12838231	12010893
		UvA	R4	15881040	15800644	14569495	13748602
		UvA	R5	16260713	16178319	14895284	14070488
		UvA	R6	15625655	15543297	14154977	13379386
	Husk	MPIPZ	R1	18019379	17928140	15963562	15128395
		MPIPZ	R2	17513029	17423799	15089641	14317370
		MPIPZ	R3	16368453	16285921	14949582	14165333
		UvA	R4	19312794	19188029	17348794	16457631
		UvA	R5	18692557	18597279	16978357	16125357
		UvA	R6	14895265	14818410	13411944	12739440

MPIPZ, Max Planck Institute for Plant Breeding Research
UvA, the University of Amsterdam
R, replicate
QC, quality control

Table S2. Number of DHSs that overlap between replicates. The numbers of overlapping peaks refer to the number of peaks in the first column that overlap with the peaks in the data sets shown at the top. Percentages relate to overlapping peaks relative to the total number of peaks indicated in the first column.

		V2-IST_1	V2-IST_2	Husk_1	Husk_2
	peaks	21309	35906	26212	17674
V2-IST_1	21309		19903	17044	13491
			93.4%	80.0%	63.3%
V2-IST_2	35906	19412		20464	15059
		54.1%		57.0%	41.9%
Husk_1	26212	16865	20666		15890
		64.3%	78.8%		60.6%
Husk_2	17674	13669	15532	16339	
		77.3%	87.9%	92.4%	

Table S3. Number of H3K9ac peaks that overlap between replicates. The numbers of overlapping peaks refer to the number of peaks in the first column that overlap with the peaks in the data sets shown at the top. Percentages relate to overlapping peaks relative to the total number of peaks indicated in the first column.

		V2-IST_1	V2-IST_2	Husk_1	Husk_1	Husk_2
	peaks	29451	21196	38206	40541	46158
V2-IST_1	29451		18336	24226	26195	27344
			62.3%	82.3%	88.9%	92.8%
V2-IST_2	21196	18427		19168	19975	20382
		86.9%		90.4%	94.2%	96.2%
Husk_1	38206	23923	18935		31696	33518
		62.6%	49.6%		83.0%	87.7%
Husk_2	40541	25675	19670	31278		37391
		63.3%	48.5%	77.2%		92.2%
Husk_3	46158	26661	20001	32713	36917	
		57.8%	43.3%	70.9%	80.0%	

Table S4. Mean frequency of GGCCCA motif per kb. Random intergenic sequences were generated using the sequence length of all candidate enhancers within each tissue.

<table>
<tr><th></th><th>Random intergenic sequences</th><th>All candidates</th><th>Candidates overlapping RLG00010 family</th><th>RLG00010 family without candidates</th><th>RLG00010 family with candidates</th></tr>
<tr><td>V2-IST</td><td>1.68</td><td>3.76</td><td>3.12</td><td rowspan="2">0.64</td><td rowspan="2">1.01</td></tr>
<tr><td>Husk</td><td>1.68</td><td>3.44</td><td>2.84</td></tr>
</table>

Supplemental methods

RNA-seq replicate library comparison

To determine the reproducibility, the RPKM values calculated using Cuffnorm [4] for each gene were plotted for every pair of replicate samples. The expression levels (in RPKM) of each gene from one replicate were plotted against normalised read counts from another replicate and the Pearson correlation coefficient was calculated using R [5].

DNase-seq and ChIP-seq library comparison

From the alignment data, peaks were called for individual libraries with a q-value cut-off of 0.001 using MACS2 [6]; then the similarities of the libraries were determined by the number of overlapping DHSs or H3K9ac enrichment peaks, which were counted using BEDtools [7], between libraries.

Asymmetric enrichment of H3K9ac around DHSs

The H3K9ac enrichment signal intensity values at 300 bp up- and downstream from the DHS boundaries were extracted using bwtool [8] and plotted as a scatter plot using R [5]. For the data sets oriented based on the H3K9ac enrichment intensities, the extracted values were sorted manually.

References

1. Jiao Y, Peluso P, Shi J, Liang T, Stitzer MC, Wang B, et al. The complex sequence landscape of maize revealed by single molecule technologies. bioRxiv. 2016;79004.

2. Regulski M, Lu Z, Kendall J, Donoghue MTA, Reinders J, Llaca V, et al. The maize methylome influences mRNA splice sites and reveals widespread paramutation-like switches guided by small RNA. Genome Res. 2013;23:1651–62.

3. Li Q, Gent JI, Zynda G, Song J, Makarevitch I, Hirsch CD, et al. RNA-directed DNA methylation enforces boundaries between heterochromatin and euchromatin in the maize genome. Proc. Natl. Acad. Sci. 2015;112:14728–33.

4. Trapnell C, Williams BA, Pertea G, Mortazavi A, Kwan G, van Baren MJ, et al. Transcript assembly and quantification by RNA-Seq reveals unannotated transcripts and isoform switching during cell differentiation. Nat. Biotechnol. 2010;28:511–5.

5. R Development Core Team. R: A Language and Environment for Statistical Computing. Vienna, Austria; 2008.

6. Zhang Y, Liu T, Meyer CA, Eeckhoute J, Johnson DS, Bernstein BE, et al. Model-based Analysis of ChIP-Seq (MACS). Genome Biol. 2008;9:R137.

7. Quinlan AR, Hall IM. BEDTools: a flexible suite of utilities for comparing genomic features. Bioinformatics. 2010;26:841–2.

8. Pohl A, Beato M. bwtool: a tool for bigWig files. Bioinformatics. 2014;30:1618–9.
